# INDETERMINATE-DOMAIN 4 (IDD4) coordinates immune responses with plant-growth in *Arabidopsis thaliana*

**DOI:** 10.1371/journal.ppat.1007499

**Published:** 2019-01-24

**Authors:** Ronny Völz, Soon-Kap Kim, Jianing Mi, Anamika A. Rawat, Alaguraj Veluchamy, Kiruthiga G. Mariappan, Naganand Rayapuram, Jean-Michel Daviere, Patrick Achard, Ikram Blilou, Salim Al-Babili, Moussa Benhamed, Heribert Hirt

**Affiliations:** 1 Center for Desert Agriculture, Division of Biological and Environmental Sciences and Engineering, King Abdullah University of Science and Technology, Thuwal, Saudi Arabia; 2 Institut de biologie moléculaire des plantes, CNRS-Université de Strasbourg 12 Rue Général Zimmer, Strasbourg cedex, France; 3 Centre National de la Recherche Scientifique, Institut National de la Recherche Agronomique, University Paris-Sud, University of Évry Val d’Essonne, University Paris Diderot, Sorbonne Paris-Cite, University of Paris-Saclay, UMR9213 Institut des Sciences des Plantes de Paris Saclay, Essonne, France; 4 Institute of Plant Sciences Paris-Saclay IPS2, CNRS, INRA, Université Paris-Sud, Université Evry, Université Paris-Saclay, Orsay, France; 5 Max Perutz Laboratories, University of Vienna, Vienna, Austria; The Ohio State University, UNITED STATES

## Abstract

INDETERMINATE DOMAIN (IDD)/ BIRD proteins are a highly conserved plant-specific family of transcription factors which play multiple roles in plant development and physiology. Here, we show that mutation in *IDD4/IMPERIAL EAGLE* increases resistance to the hemi-biotrophic pathogen *Pseudomonas syringae*, indicating that *IDD4* may act as a repressor of basal immune response and PAMP-triggered immunity. Furthermore, the *idd4* mutant exhibits enhanced plant-growth indicating IDD4 as suppressor of growth and development. Transcriptome comparison of *idd4* mutants and *IDD4*ox lines aligned to genome-wide IDD4 DNA-binding studies revealed major target genes related to defense and developmental-biological processes. IDD4 is a phospho-protein that interacts and becomes phosphorylated on two conserved sites by the MAP kinase MPK6. DNA-binding studies of IDD4 after flg22 treatment and with IDD4 phosphosite mutants show enhanced binding affinity to *ID1* motif-containing promoters and its function as a transcriptional regulator. In contrast to the IDD4-phospho-dead mutant, the IDD4 phospho-mimicking mutant shows altered susceptibility to *PstDC3000*, salicylic acid levels and transcriptome reprogramming. In summary, we found that IDD4 regulates various hormonal pathways thereby coordinating growth and development with basal immunity.

## Author summary

This work illustrates the involvement of the IDD family member 4 in the regulation of defense responses against hemibiotrophic pathogens and in processes governing plant growth. IDD4 is embedded in widely-ramified regulatory pathways and exerts transcriptional control of key factors that shape and balance growth with defense. Mutation in *IDD4* and overexpression affect the plant shoot and root growth. At the same time, *IDD4* acts as a repressor of innate immunity against the hemi-biotrophic pathogen *Pst DC3000*. IDD4 interacts with and is *in vitro* phosphorylated by the immune MAP kinase MPK6. Genome-wide IDD4 DNA-binding studies revealed subsets of direct targets contributing to various developmental processes and immunity. Chromatin-immunoprecipitation and DNA-shift experiments identified the *ID1* motif as the prime target site of IDD4 and revealed that phospho-modifed IDD4 shows altered DNA binding ability and thereby gene expression and pathogen resistance. Overall, these results indicate that IDD4 links signal transduction to gene expression of genes involved in basal immunity and growth and development.

## Introduction

Plants and animals use pattern recognition receptors (PRRs) to rapidly activate defense signaling pathways and immune responses upon pathogen attack [1, 2]. PRR receptors and their associated signaling components possess a wide range of similarities in mammals, plants and invertebrates [3, 4]. Plant immunity relies on the recognition of pathogen-derived molecules in order to activate pattern-triggered immunity (PTI) and effector-triggered immunity (ETI) [5]. PTI is initiated after the perception of pathogen-associated molecular patterns (PAMPs) of highly conserved pathogen components. FLAGELLIN22 (flg22), a 22 amino acid peptide from within bacterial flagellin protein, represents one important PAMP to trigger PTI in plants and is perceived by the plasma membrane-localized receptor FLAGELLIN-INSENSITIVE2 (FLS2) which associates with BRI1-ASSOCIATED RECEPTOR KINASE (BAK1) in order to rapidly stimulate two mitogen-activated protein kinase (MAPK) cascades. MAPK cascades consist of three sequentially activated kinase modules composed of a MAPK kinase kinase, a MAPK kinase and eventually a MAPK, thereby linking upstream signals to downstream targets. In *Arabidopsis* as well as throughout the plant kingdom, the MAPK orthologues of MPK3, MPK4 and MPK6 represent the final step in the two flg22-activated MAP kinase cascades and transmit signals to respective target proteins by phosphorylation [6, 7]. MPK3, MPK4 and MPK6 are required for full activation of defence genes [8]. In particular, MPK3/MPK6 contributes to bacterial and fungal resistance [9] as well as to a multitude of developmental processes, including the regulation of plant architecture, seed, root [10] and stomatal formation [11]. Furthermore, the MPK6 signaling module participates in nutrient signaling to influence nitrate assimilation, enhances phosphate acquisition and becomes activated upon iron deficiency [12]. Recently, it was shown that MPK3/MPK6 exert essential functions in the induction of camalexin, the major phytoalexin in *Arabidopsis*, and promote the indole glucosinolate biosynthesis pathway [9]. In addition, MPK3/MPK6 activation rapidly alters the expression of photosynthesis-related genes and inhibits photosynthesis, which promotes the accumulation of superoxide(O_2_) and hydrogen peroxide (H_2_O_2_), two major reactive oxygen species (ROS), in chloroplasts under light [13].

The *INDETERMINATE DOMAIN* (*IDD*)/*BIRD* family of transcription factors (TF) is highly conserved in both monocots and dicots and functions in multiple developmental processes [14, 15]. IDDs are a plant-specific group of TF comprising of 16 members in *Arabidopsis* which are characterized by a conserved N-terminal ID domain composed of four zinc fingers (ZFs) and a long undetermined sequence for protein interaction [16]. The four ZFs can be subdivided into the C_2_H_2_ type ZF1 and ZF2, which are dedicated to DNA interaction, and the C_2_HC type ZF3 and ZF4. ZF3 and in particular ZF4 are essential for the interactions of IDD3/MAGPIE and IDD10/JACKDAW to the SHORT-ROOT (SHR)—SCARECROW (SCR) complex. By contrast, ZF1-ZF2-ZF3 of IDD3 and IDD10 are involved in DNA binding [17]. In *Arabidopsis*, IDD4/IMPERIAL EAGLE functions in ad-/abaxial leaf development and leaf blade formation and its expression is subject to KANADI1 and the HD-ZIPIII family protein REVOLUTA. [18]. Furthermore, among other IDD family members, IDD4 contributes to the root ground tissue organisation and coordinates the differentiation of the endodermis initial stem cell niche in order to give rise to cortex and endodermis cells [16, 19, 20] and serves as transcriptional scaffold to enable transactivation activity of the gibberellin-inhibitor DELLA/RGA proteins of the GRAS-family in association with the transcriptional regulator SCARCROW-like 3 (SCL3) [16, 21, 22].

Several independent studies identified IDD4 to be phosphorylated on serine-73, a highly conserved putative MAPK motif that is conserved in all family members of the IDD/BIRD family [23–29]. Moreover, using an inducible MAPKK-activation system, Ser73 [29] was identified as a target of the MAPKs MPK3 and MPK6. These studies prompted us to test IDD4 as putative regulator and MAPK substrate in plant innate immunity. Transcriptome and global ChIP-SEQ analysis of *idd4 and IDD4ox* plants revealed that IDD4 plays a role in coordinating innate immunity with growth and development. ChIP-qPCR analysis showed that flg22-treatment correlates with the recruitment of IDD4 to *ID1* motif-containing promoter regions. Moreover, IDD4 interacts and becomes phosphorylated by the immune MAPK MPK6, and that IDD4-phosphomimichking versions show enhanced DNA-binding and transcriptional activity of *ID1* motif-containing promoters. Phosphosite-mutated IDD4 plants show opposite susceptibility to pathogen attack and transcriptome reprogramming, confirming the function of IDD4 in regulating genes related to immunity and plant-growth.

## Results

### Expression analysis of *IDD4*

**Fig 1:**
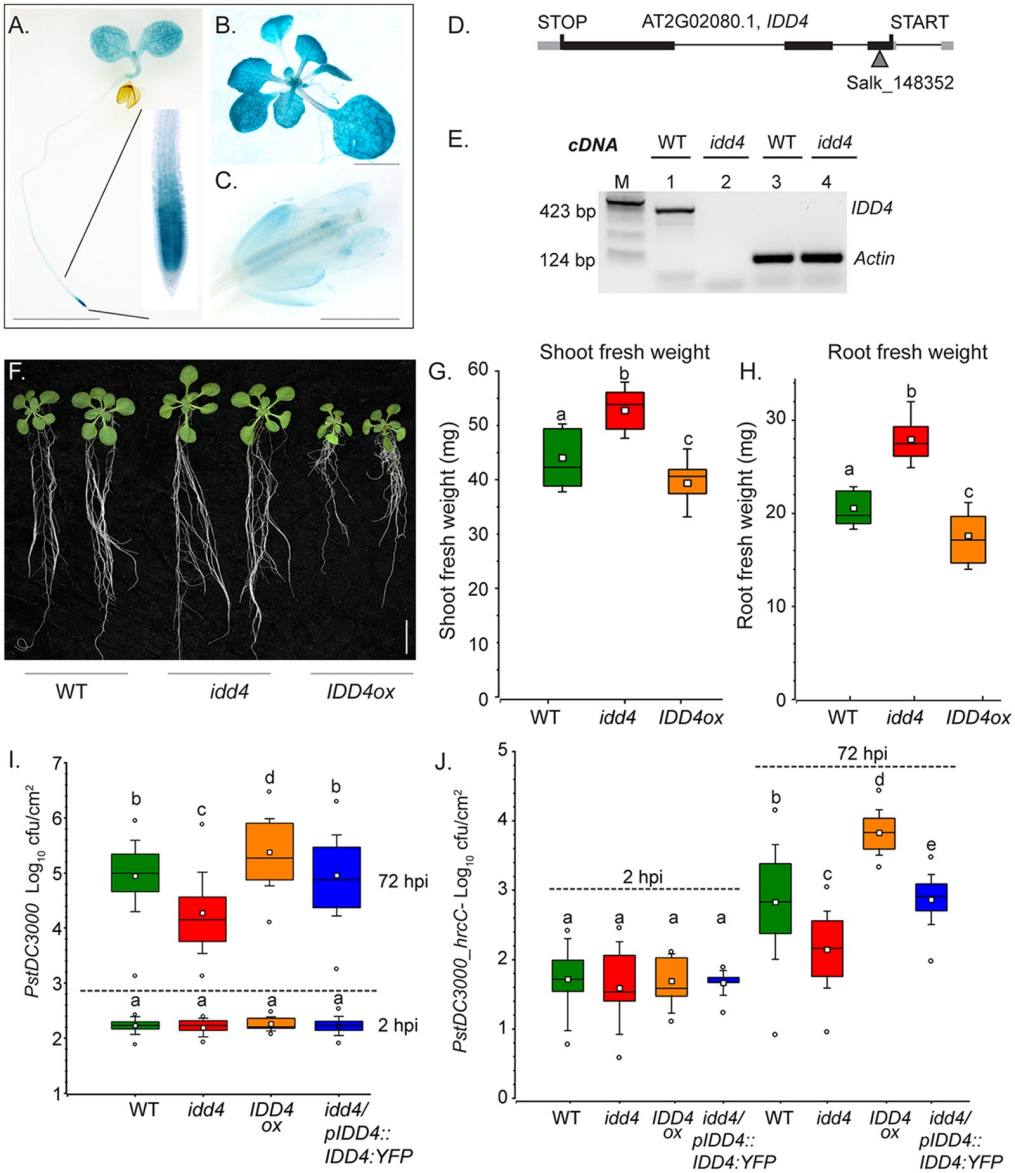
Expression analysis of IDD4 and functional characterization of idd4 mutants. **(A-C)** *pIDD4*::*GUS* reporter lines under the control of the 2.5kb upstream region of the translational start sequence of *IDD4* showing GUS staining in the cotyledons, hypocotyl, root tip **(A)**, as well as in rosette leaves **(B)** and flower organs **(C)** (see also S1 Fig) Scale bar = 2.5 mm. **(D)** Genomic structure of *IDD4* locus showing the position of T-DNA insertion. The *idd4* mutant allele resulting from the T-DNA insertion in the 1^st^ exon was confirmed to be homozygous (see also S2A Fig). **(E)** RT-PCR based *IDD4* transcript evaluation in *idd4* compared to wild-type (WT) by using oligo-nucleotides 352s/as, loading control c*ACTIN* P107/108 (S7 Table). **(F)** Phenotype of 18 day-old WT compared to *idd4* mutant plants and gain-of-function mutant *IDD4ox1* (*pUBI10*::*GFP*:*IDD4*), cultivated on Murashige and Skoog basal medium under long-day conditions. Scale bar = 10 mm. **(G-H)** Fresh weight of shoot and root of 18 day-old WT plants compared to *idd4* and *IDD4ox* lines. Boxes represent the 25th and 75th percentiles and the inner rectangle highlights the median, whiskers show the SEM, letters above boxes represent significance groups as determined by multiple comparison Student’s test *p*≤0.01. Plants of three biological replicates (*n* = 30) were analysed (see also S2B–S2E Fig). **(I-J)** WT plants, *idd4* mutant, *IDD4ox* line and *idd4* complementation line (*pIDD4*::*IDD4*:*YFP*) were treated by *PstDC3000* **(I)** and *PstDC3000 hrcC-* **(J)**. Boxes represent the 25th and 75th percentiles and the inner rectangle highlights the median, whiskers show the SEM, and outliers are depicted by dots (Min/Max range), letters above boxes represent significance groups as determined by multiple comparison Student’s test *p*≤0.05. Plants of three biological replicates (*n* = 30), were spray-inoculated with a bacterial suspension of OD_600_ 0.2, the density of colony-forming units (cfu) was analyzed 2 and 72 hours post inoculation (hpi).

So far, it was reported that IDD4 is expressed in the root ground tissue of the basal meristem [20]. To investigate the contribution of IDD4 in defense response, its expression was analysed by generating stably transformed *Arabidopsis* lines expressing *GUS* or *NLS*:*3xGFP* under a 2.5 kb *IDD4* promoter sequence. Intense staining of the GUS reporter was observed in cotelydons, root tips and in all stages of rosette leaf development (Fig 1A and 1B). In leaves, we detected GUS/GFP signals in the trichomes, stomata, epidermal cells (S1A–S1D Fig) and mesophyll cells (S1E Fig). Moderate GUS staining was observed in sepals and petals (Fig 1C) as well as in ovules embedded in carpels of *Arabidopsis* flowers (S1F Fig). Various public microarray datasets (Genevestigator) (S1G Fig) showing endogenous *IDD4* transcript abundance corresponds to our histological results and reveal the expression of *IDD4* in a wide range of tissues throughout the life cycle in *Arabidopsis*.

### Enhanced plant-growth and pathogen-resistance of *idd4* mutants

The functional contribution of IDD4 in response to bacterial pathogen attack was investigated by challenging *idd4* mutant, a complementation line *(idd4/pIDD4*::*IDD4*:*YFP*) and an overexpressor line *IDD4ox1* (*pUBI10*::*GFP*:*IDD4*) with the virulent hemi-biotrophic plant pathogen *Pst DC3000*. We used an *IDD4* insertion line (Salk_148352) containing the T-DNA insertion in the first exon that could be confirmed by sequencing as a true knockout line (Fig 1D and 1E and S2A Fig). The *idd4* complementation line expresses *IDD4*:*YFP* driven by the 2.5 kb upstream sequence of the *IDD4* ORF. Interestingly, by determining the fresh weight of 18 day-old plants, we consistently found an increase of approximately 20% biomass in the aerial part of the *idd4* mutant. By contrast, the shoot fresh weight of the four analyzed *IDD4ox* lines showed a reduction of about 11.5% in *IDD4ox1* (Fig 1F and 1G) up to approximately 65% in *IDD4ox4* when compared to WT (S2B–S2D Fig). Interestingly, the growth reduction seems to be in accordance with the ectopic expression of *IDD4* in *IDD4ox1-4* lines (S2B Fig). The differences in shoot growth also corresponded to altered root formation in the *idd4* and *IDD4ox* lines. The root biomass in *idd4* is increased by about 36% whereas that of the *IDD4ox* lines show a reduction of approximately 14% (Fig 1F and 1H) in *IDD4ox1* and 75% in *IDD4ox4* (S2C and S2D Fig). The enhanced growth of *idd4* could be reverted to WT by the expression of the complementation construct (S2E Fig). The restored phenotype in *idd4/pIDD4*::*IDD4*:*YFP* shows that the improved growth can be traced back to the mutation in *IDD4*. The differences in shoot and root growth in *idd4* and *IDD4ox* lines suggest a function of IDD4 as a regulator of growth-associated processes.

Two hours after spray infection by *PstDC3000*, the infection levels in the different transgenic lines corresponded to those in WT plants indicating that stomatal immunity was not affected (Fig 1I). However, 72 hours after spay-infection, the proliferation levels of *Pst DC3000* in the *idd4* mutant were significantly reduced when compared to WT. Furthermore, the bacterial titer in the *idd4* complementation line was indistinguishable from WT, suggesting that the reduced susceptibility in *idd4* mutants is due to the lack of IDD4 protein function. By contrast, the *IDD4ox* line exhibited increased susceptibility to *Pst DC3000* (Fig 1I). Therefore, we reasoned that IDD4 acts as a negative regulator of basal resistance to hemi-biotrophic pathogen infection. To evaluate the PTI response, the *idd4* mutant was challenged by *Pst DC3000 hrcC-*. The *Pst DC3000 hrcC-* strain is compromised in virulence due to its inability to inject any of its type III-secretion system-dependent effectors, one function of which is to suppress plant immunity. In this way, infection with *Pst DC3000 hrcC-* principally induces only PTI-mediated defense responses. In comparison to WT plants and *idd4* complementation lines, the proliferation levels of the bacteria 72 hrs after spray-infection were reduced in *idd4* mutants and elevated in *IDD4ox* (Fig 1J). The higher resistance of the *idd4* mutant indicates an enhanced PTI response thereby suggesting that IDD4 also functions to regulate PTI-mediated defense responses.

### IDD4 regulates genes that coordinate specific processes in plant growth and immunity

**Fig 2:**
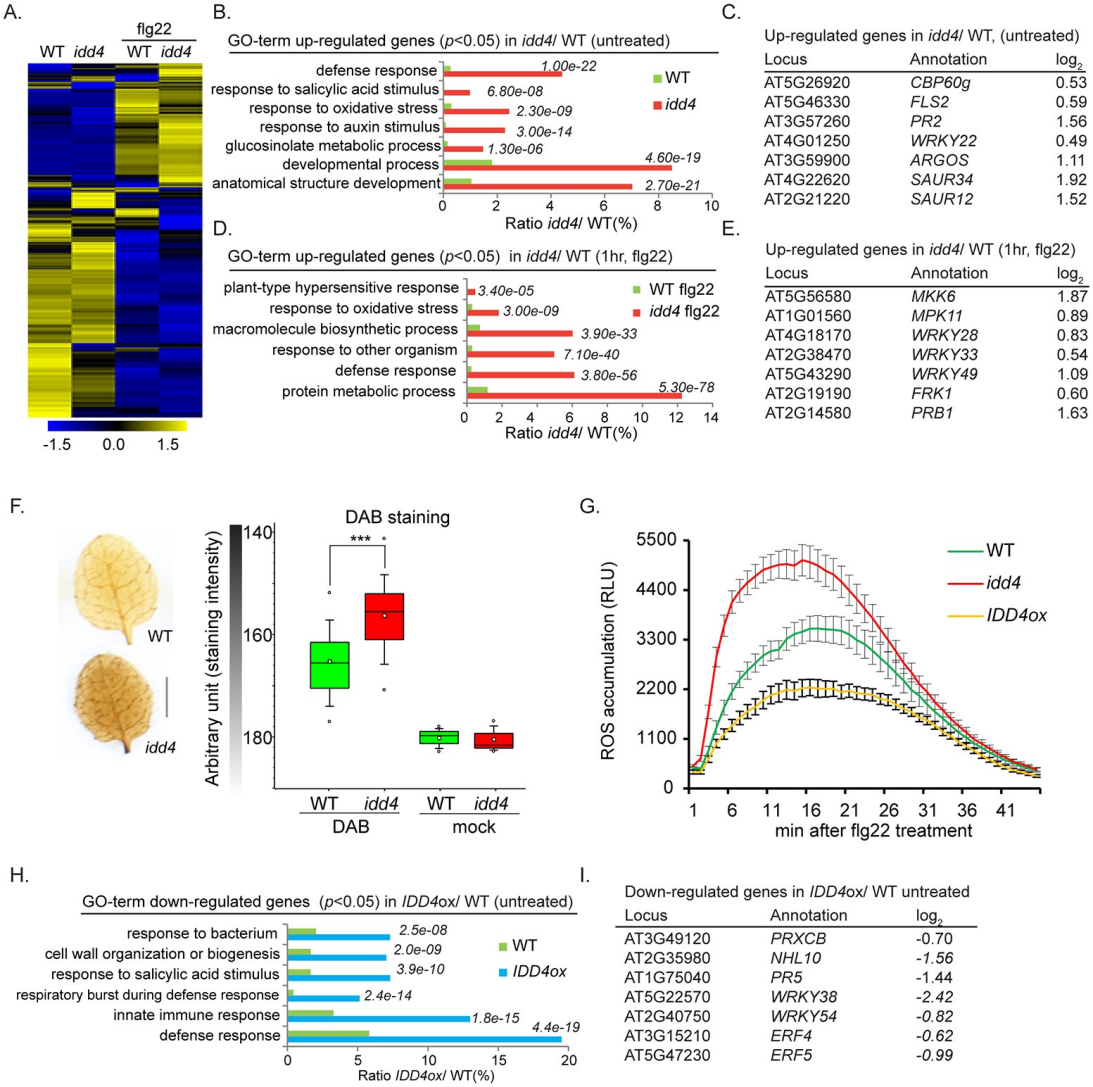
Transcriptome composition of *idd4*, *IDD4ox and idd4* flagellin22-treated lines associated with the characterization of pathogenicity-associated traits. **(A)** Transcriptome comparison of WT and *idd4* mutant with and without flg22 treatment. The original FPKM values were adjusted by normalized genes/rows and subsequently processed by hierarchical clustering by means of average linkage method using MeV4.0. Blue and yellow color indicates relatively low and high expression levels, respectively. For complete gene list see S1 Table. **(B-E)** Transcriptome composition and gene ontology annotations of up-regulated genes (*p*<0.05) in *idd4* mock **(B-C)** and *idd4* flagellin22 treated samples **(D-E)**, GO terms were determined by using the AgriGo database (TAIR9, genomic locus). For complete gene list see S2 Table. **(F)** Evaluation of H_2_O_2_ levels by 3,3'- diaminobenzidine staining (DAB) in untreated *idd4* mutants compared to WT. Scale bar = 2.75 mm. Boxes represent the 25th and 75th percentiles and the inner rectangle highlights the median, whiskers show the SEM, and outliers are depicted by dots (Min/Max range). Statistical significance was analyzed by Student’s test, asterisks indicate significant difference, ***p<0.001. **(G)** flg22-induced ROS burst assay of *idd4*, *IDD4ox* and WT plants, 5 week old plants were treated with 1 μM flg22 treatment for 45 min, values indicate mean ± SE, *n* = 36 (3 biological replicates). **(H-I)** Transcriptome composition and gene ontology annotations of down-regulated genes (*p*<0.05) in *IDD4* gain-of-function mutant (*pUBI10*::*GFP*:*IDD4*). GO terms were determined by using the agriGo database (see also S2 Table).

In order to analyse the transcriptome composition of *idd4* and *IDD4ox* lines, we performed *RNA*-Hiseq analysis on 3 biological replicates of 14 day-old *idd4*, *IDD4ox* and WT seedlings without and after flg22 application (1μM flg22, 1hr). A close to linear correlation coefficient of WT and *idd4* (0.85), WT and *idd4* (flg22) (0.87), as well as WT and *IDD4ox* (0.98) was obtained when considering the expression profiles for all transcripts. The strict correlation suggests that IDD4 does not affect general gene expression, but rather influences subsets of genes in particular biological processes. Hierarchical clustering of significant genes (*p*<0.05) in *idd4* before and after flg22 treatment, by using normalized FPKM values, revealed distinct differences in gene expression patterns suggesting altered gene induction in *idd4* after flg22 perception (Fig 2A, S1 Table). At a stringency of *p*<0.05 in untreated *idd4* mutants, 2244 differentially expressed genes (DEGs) (S2 Table) could be identified that show a log_2_-fold change from 0.27 (1.21 FC) to 4.05 (16.57 FC) of positively regulated genes and from -4.95 (30.88 FC) to -0.27 (1.21 FC) of negatively regulated genes. Among these 2244 genes, 621 genes are up- and 1623 genes are down-regulated. To categorize DEGs in functional modules, gene ontology (GO) terms were determined by using the AgriGO platform [30] (TAIR9) (Fig 2B, S2 Table). The up-regulated genes can be grouped in very different GO terms describing gene functions in different hormonal pathways and a multitude of cellular biological processes. GO terms are highlighted for defense response, response to salicylic acid stimulus, oxidative stress and response to other organism thereby indicating a function of IDD4 in the repression of defense-related genes and factors contributing to growth and development. Intriguingly, transcript levels of *CALMODULIN-BINDING PROTEIN 60g* (*CBP60g)* [31] that regulates expression of the rate-limiting enzyme *ICS1* in SA biosynthesis [32] and the SA marker gene *PATHOGENICITY-RELATED FACTORS PR2* were expressed at significantly higher levels in untreated *idd4* plants compared to WT (Fig 2C and S2F Fig). Similarly, enhanced expression was found in *idd4* mutant plants for the pattern-triggered immunity-responsive marker gene *FLG22-INDUCED RECEPTOR-LIKE KINASE 1* (*FRK1*) [33], and the early-defense marker transcription factor *WRKY22* (Fig 2C and S2F Fig).

Interestingly, the enhanced growth phenotype of the *idd4* mutant corresponds with GO terms describing gene functions for response to auxin stimulus, glucosinolate metabolic process and anatomical structure development. The enrichment of auxin response genes, including *AUXIN-REGULATED GENE INVOLVED IN ORGAN SIZE (ARGOS)* and several members of the SMALL AUXIN UPREGULATED RNA (SAUR)-like auxin-responsive protein family (Fig 2C, S2 Table), promoting plant growth and architecture [34], in *idd4* mutant plants might partly explain its elevated biomass. In addition, the *idd4* transcriptome was enriched in genes of the glucosinolate metabolism that participates both in defense and growth [9, 35]. After flg22 treatment, 2048 DEG (*p*<0.05) were obtained in *idd4* mutant, with 1063 genes showing enhanced while 985 genes reduced transcript abundance (S2 Table). In accordance with the untreated *idd4* transcriptome, the GO analysis of up-regulated genes after flg22 application emphasized GO terms for hypersensitive response, oxidative stress and defense response (Fig 2D). In particular, the expression of the defense markers *WRKY33*, *WRKY49* and *FRK1* is elevated as well as components of the kinase signaling cascades represented by *MKK6* and *MPK11* (Fig 2E). Additionally, macromolecular biosynthesis process and protein metabolic process are among the most significant GO terms (Fig 2D, S2 Table).

By contrast to the *idd4* mutant, the overexpression of *IDD4* (*IDD4ox*) reduces defense-related gene expression depicted by the GO analysis of significantly down-regulated genes (*p*<0.05) (S2 Table). These DEGs are functionally grouped in GO terms for respiratory burst during defense response, salicylic acid stimulus and innate immune response (Fig 2H). Prominent representatives are *WRKY38* [36], *PR5* as well as *ERF4* and *ERF5* [37] (Fig 2I). The deregulation of genes and functional groups dedicated to immunity in the *idd4* mutant and *IDD4ox* lines confirms the role of IDD4 as a regulator of defense-related gene expression.

In accordance, the GO analysis before and after flg22 treatment show DEGs involved in the response to reactive oxygen species (ROS) which belong to the first line of defense upon pathogen invasion [38]. Intriguingly, genes coding for the H_2_O_2_-scavenging enzymes CATALASE 1 (CAT1) and CAT2, which catalyze the reduction of photo-respiratory generated hydrogen peroxide and protect cells from its toxicity [39], are down-regulated in *idd4* (S2 Table). Interestingly, SA-mediated suppression of CAT2 results in increased H_2_O_2_ levels and resistance against biotrophic pathogens [40]. Furthermore, *PEROXIDASE CA* (*PRXCA*) [41], *ASCORBATE PEROXIDASE 1* (*APX1*) [42] and *L-ASPARTATE OXIDASE* (*AO*) [43] are up-regulated after flg22-treatment in *idd4* and have been reported to promote H_2_O_2_ metabolism and turn-over. By comparing the levels of the ROS H_2_O_2_ by 3,3'-diaminobenzidine (DAB) staining in untreated *idd4* with WT plants, we observed that the H_2_O_2_ levels in *idd4* were strongly elevated already before infection (Fig 2F). In the mock-treated control without applied DAB, the staining value is about 180 of an arbitrary unit for WT and *idd4* and can be considered as default level. After DAB staining, WT-plants showed a staining value of 165 and *idd4* of about 156. This staining difference indicates a higher H_2_O_2_ accumulation in the *idd4* mutant. Additionally, in a comparative flg22-triggered reactive oxygen species burst assay, we detected strikingly enhanced ROS levels in *idd4* mutants and reduced ROS efflux in *IDD4ox* lines when compared to WT (Fig 2G). Notably, maximum ROS levels were observed in WT at 17 minutes whereas the *idd4* mutants achieved maximal ROS production already at 7 minutes after flg22 perception. These results show that the GO term annotations response to oxidative stress in *idd4* mutant and respiratory burst during defense response in *IDD4ox* plants correspond with their biochemical traits. In summary, the constitutive activation of ROS and defense-related traits and the enrichment of factors that promote structural development indicate a role of IDD4 in growth and immunity-related processes.

### Primary target genes of IDD4 contribute to growth and defense-associated processes

**Fig 3:**
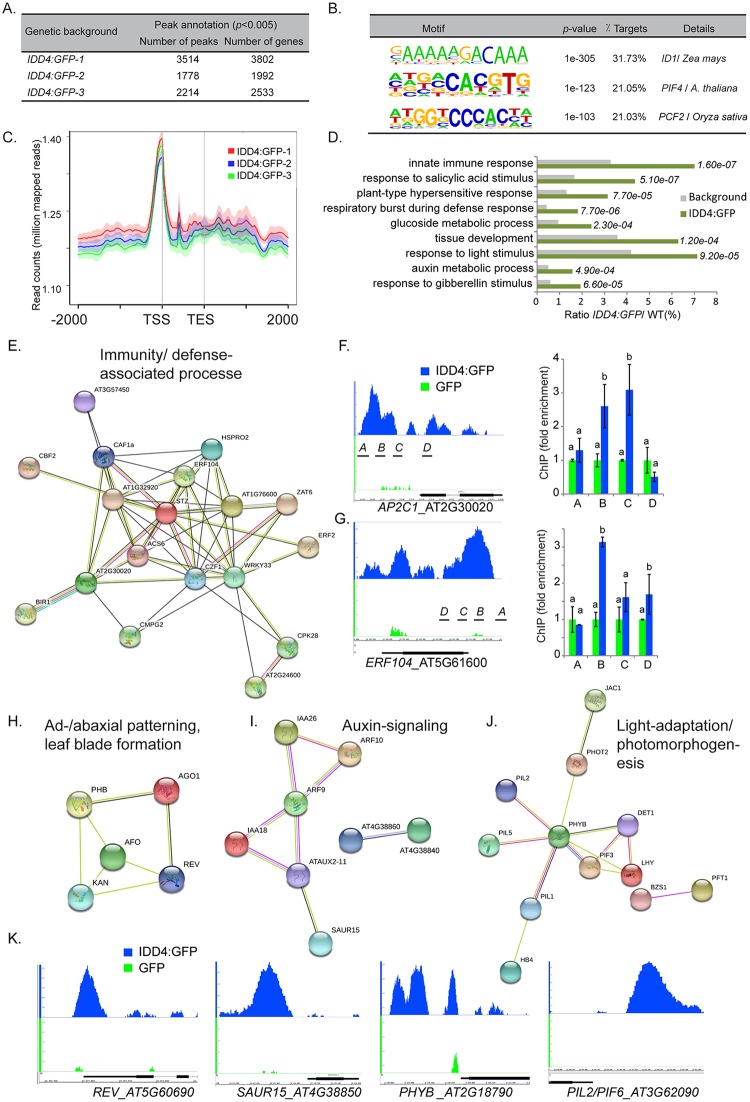
Genome-wide identification of IDD4 chromatin-bound regions and functional categorization of primary target genes. (**A**) The number of significant peaks per biological replicate with an FDR <0.005 and the number of gene annotations is depicted for the independent biological samples. Qualitative evaluation of the single biological replicates was performed by co-occurance matrix generation shown in S3C Fig. (**B**) IDD4 target sequences share common DNA-binding motifs. The most frequent site consists of the core motif AGACAA (*ID1* motif) and can be found in 31.73% of IDD4 targets (*p* = 1e-305). (**C**) Mean profile of IDD4 ChIP-SEQ read density of three independent biological samples within the ±2 kb region with respect to a gene model from transcriptional start sequence (TSS) to transcriptional end sequence (TES). Predominant binding of IDD4 in the region 500bp upstream of the TSS could be observed. (**D**) Gene ontology annotations of significant ChIP-SEQ targets (S3 Table) (**E, H-J**) Protein interaction networks derived from the IDD4 ChIP-SEQ targets. All significant IDD4 ChIP-SEQ targets were pooled and used to generate a network using STRING (version 10.0). Minimum required interaction score defined as high confidence 0.700, Meaning of network edges “evidence”. (**F-G**) Binding profiles of IDD4 to the *AP2C1* (**F**) and *ERF104* (**G**) loci. ChIP-SEQ profiles and ChIP-qPCR evaluations are depicted for each locus. The TAIR annotations of the genomic loci are shown at the bottom of each panel. The genomic locus indicated above the scale represents forward (+) orientation, while the one below represents reverse orientation. In each case, the enrichment was found to be in the upstream region of the respective genomic locus. (**K**) Binding profiles of IDD4 to the *REV*, *SAUR15*, *PHYB* and *PIF6* loci. The TAIR annotation of the genomic loci is shown at the bottom of each panel. The genomic locus indicated above the scale represents forward (+) orientation, while the one below represents reverse orientation. In every case, the enrichment was found to be in the upstream region of the respective genomic locus.

In order to identify the genomic regions targeted by IDD4 *in vivo*, Chromatin-Immunoprecipitation (ChIP) was performed followed by deep sequencing on the Illumina High-Sequencing platform (SEQ). In order to evaluate the reproducibility of the obtained genome-wide IDD4 binding profiles, ChIP-SEQ was performed on 3 independent lines carrying the *IDD4*:*GFP* construct under the control of the *UBI10* (At4g05320) promoter. The retrieved binding patterns were normalized by comparison with data obtained from *pUBI10*::*GFP* plants. GFP antibodies were used to immunoprecipitate protein-DNA complexes that were verified by Western-Blot analysis before sequencing (S3A Fig). Sequence coverage at each position on the genome was plotted to identify peaks in the *Arabidopsis* genome. A significant number of peaks per biological replicate with an FDR <0.005 could be annotated (Fig 3A) in the 500 bp region upstream of the translational start sequence in the genic region. A highly consistent read pattern in all three *IDD4*:*GFP* lines showed predominate IDD4 binding in the vicinity upstream of the transcriptional start site (Fig 3C), which includes the 5’-UTRs and promoter regions. Sequence analysis of the 500 bp regions upstream of the TSS of IDD4 targets revealed the *in vivo* enrichment of the *ID1* motif on a genome-wide scale in about 31% of target genes (Fig 3B) and was ranked as the primary binding site. Previously, the core sequence of the *ID1* motif (AGACAA) was initially suggested as binding site of the maize ID1 protein [44], and further *in vitro* characterized for its association with IDD3 and IDD8 in *Arabidopsis* [16, 45]. To compare the independent transgenic lines, the binding of IDD4:GFP to the same target sequences was monitored by co-occurrence matrix analysis (S3C Fig). The comparison illustrates that replicates 1 and 3 provide the most reliable overlapping binding sites. After removing genes that showed similar sequence peaks in the negative control, 837 genes could be defined as significant binding targets of IDD4 (S3 Table).

A GO term analysis of the 837 major IDD4 target genes highlighted gene functions in innate immune response, response to salicylic acid stimulus, hypersensitive response and respiratory burst during defense response (Fig 3D). These GO terms demonstrate that a significant part of the direct IDD4 targets are assigned to functions in defense response and pathogenicity (S3 Table). On the other hand, IDD4 exhibits binding preferences on genes that mediate cellular processes governing growth and development, including auxin and glucoside metabolic process as well as response to gibberellin and light stimulus. To gain a more precise view on global IDD4 function, target genes were grouped into protein interaction networks by performing gene cluster analyses using STRING [46]. Versatile functional networks could be generated with IDD4 in various distinct biological processes (Fig 3E and 3H–3J). Several genes are integrated into a ramified functional network that contributes to defense-associated processes (Fig 3E), including *WRKY33*, whose phosphorylation by MPK6 promotes the transcriptional induction of camalexin biosynthesis genes [47]; *ERF104*, a key regulator of basal immunity whose stability and interaction with MPK6 is compromised in response to flg22 [48]; *AP2C1*, which interacts and dephosphorylates MPK6 and consequently suppresses immunity [49]; *CAF1a*, a mRNA deadenylase targeting transcripts for post-transcriptional modification with temporal specificity during plant defense response [50], and *BIR1*, encoding a BAK1-interacting receptor-like kinase and activates plant defense responses [51]. IDD4 binding profiles of *AP2C1* and *ERF104* generated by ChIP-SEQ and ChiP-qPCR confirmed the association of IDD4 to the 5’upstream region of the respective gene loci (Fig 3F and 3G). The association of IDD4 to these target genes indicates its direct influence on factors shaping immunity response (Fig 3E).

Furthermore, a prominent hub describes IDD4 function in the ad/abaxial pattern specification and leaf blade formation by regulating *REVOLUTA* (*REV*), *KANADI* (*KAN*), *PHABULOSA* and *ABNORMAL FLORAL ORGANS* (Fig 3H and 3K) [18]. A further functional node combining factors involved in auxin-signaling was formed and comprises the *AUXIN RESPONSE FACTOR ARF9* and *ARF10*, and the *INDOLE-3-ACETIC ACID INDUCIBLE IAA18* and *IAA26*, as well as one member of the *SAUR* family, suggesting a regulatory role of IDD4 in auxin-mediated growth processes (Fig 3I and 3K). Moreover, recently published data indicate IDD4 as a transcriptional activator of nuclear-encoded photosynthetic gene expression and photomorphogenesis [52]. In this context, chloroplast maturation and import machinery, as well as chlorophyll biogenesis, seems to be targeted by IDD4 (Fig 3J), as shown by the binding to the promoter regions of *PHYTOCHROME B*, *PHYTOCHROME INTERACTION FACTOR* (*PIF) 3*, *PIL1*, *PIL2/PIF6*, *PIL5/PIF1* [53] (Fig 3K) and *Chloroplast heat shock protein 70–1* (*cpHsc70-1*) [54] together with *DE-ETIOLATED 1* (*DET1*) [55]. Moreover, *RNA POLYMERASE SIGMA SUBUNIT 2* (*SIG2*) [56] encodes a subunit of chloroplast RNA polymerase, and *REDUCED CHLOROPLAST COVERAGE 2 (REC2)* [57] which contributes to the size establishment of the chloroplast compartment are two of several further IDD4 target genes that are involved in the chloroplast biogenesis and function (S3 Table).

**Fig 4:**
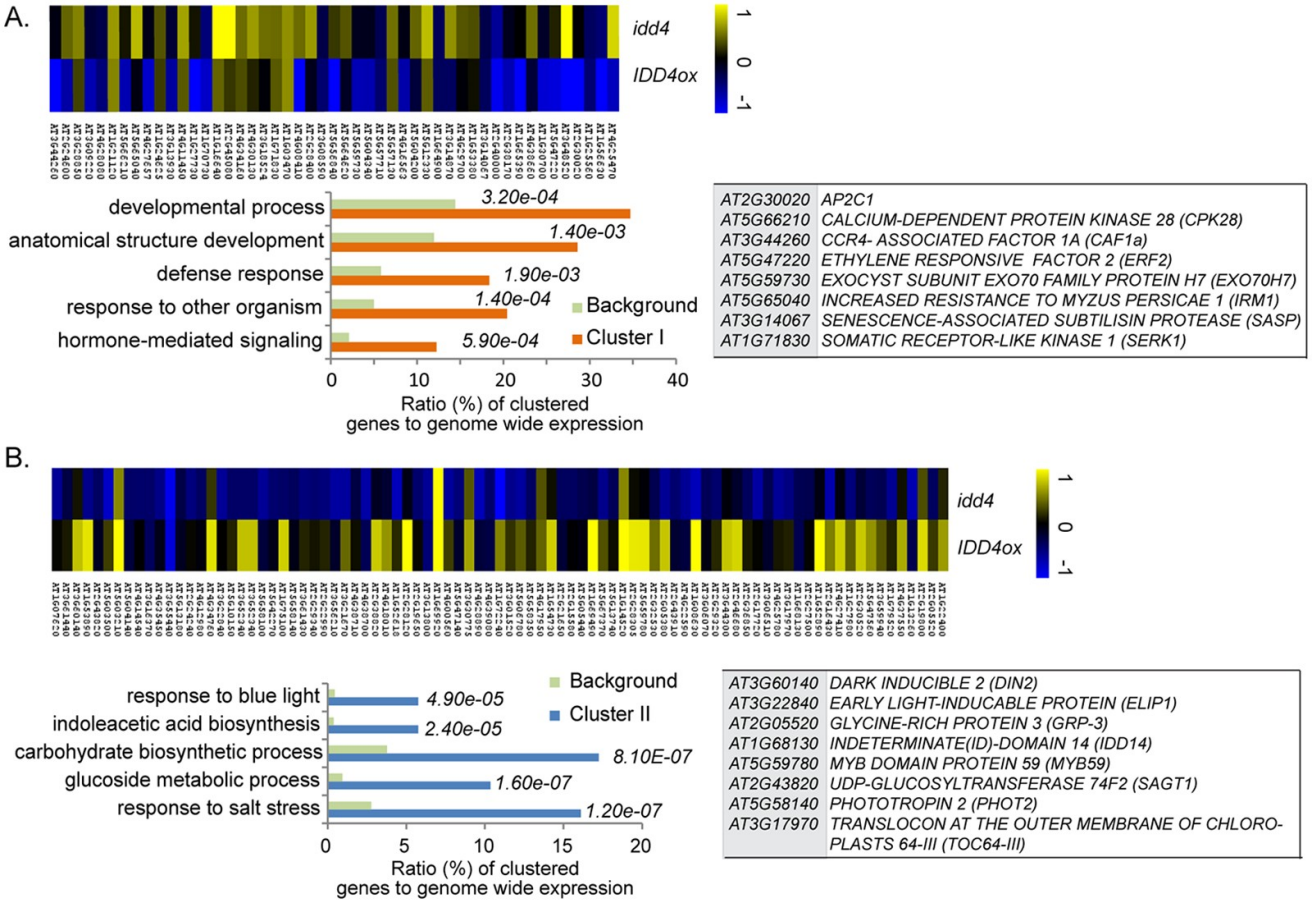
Hierarchical clustering of IDD4 target gene collection, differentially regulated in *idd4* mutant and *IDD4ox* lines. (**A**) Cluster I shows GO annotations and selected IDD4 target genes being predominately up-regulated in *idd4* mutant and/or down-regulated in *IDD4ox* lines. (**B**) Cluster II displays aligned IDD4 target genes that are prevalently down-regulated in *idd4* mutant and/or up-regulated in *IDD4ox* line; furthermore, GO annotations and selected genes are presented. (**A-B**) Log_2_ (FC) (*p*<0.05, *idd4/WT*, *IDD4ox/WT*) of individual genes was used for clustering by using the average linkage method under Pearson Correlation (MeV4.0) (for complete gene list see S4 Table).

To pinpoint whether IDD4 acts as a positive or negative regulator of major ChIP-SEQ target-genes, we compared DEGs identified in the transcriptome analysis of the *idd4* mutant and *IDD4* overexpression lines. The comparison of CHIP-SEQ targets with DEGs in *idd4* and *IDD4ox* revealed an overlap of 11.7% and 6.7%, respectively. Altogether, the search for direct targets yielded 135 genes (S4 Table) that can be grouped by GO terms for glucoside metabolic process (*p* = 1.00e^-08^), defense (*p* = 1.10e^-06^) and immune response (*p* = 8.10e^-05^). The predominantly opposite regulation of these target genes in the two genetic backgrounds suggests that IDD4 functions as a direct regulator of the respective genes. In this regard, hierarchical clustering of differentially regulated major targets yielded 49 genes matching the criteria of being predominantly up-regulated in *idd4* and down-regulated in *IDD4ox* which indicates IDD4 as transcriptional repressor of this cluster (Fig 4A, S4 Table). The corresponding GO terms emphasise hormone-mediated signaling, response to other organism, defense response and developmental process. In addition, transcriptionally repressed targets of IDD4 are involved in various defense processes including *AP2C1*, *CPK28*, *CAF1a* and *SERK1*, thereby indicating IDD4 as a direct regulator of genes in immunity. STRING-based protein interaction network of these 49 genes created clusters combining target genes involved in immunity like *AP2C1*, *CAF1a*, *CPK28* and *ERF2* with those involved in vesicle-transport, like EXO70H7 [58] (S5A Fig).

On the other hand, 86 genes identified in the DEGs and CHIP-SEQ overlap predominantly correspond to down-regulated genes in *idd4* but enhanced expressed in the *IDD4ox* lines (Fig 4B, S4 Table). The selected genes can be grouped in GO terms for response to blue light, response to salt and carbohydrate biosynthesis process. Furthermore, the supported GO terms indoleacetic acid biosynthesis, and glucoside metabolic process refer to IDD4 function as a regulator of genes that participate in growth and development. Gene cluster analyses using STRING created several functional modules (S5B Fig) for light response/ photorespiration/ photo-morphogenesis and chloroplast import machinery emphasizing again the contribution of IDD4 in processes mediating growth and development. Noteworthy, the expression of *IDD4* is compromised by defects in the chloroplast import machinery and the retrograde transport, and it is postulated to act as a transcriptional activator of nuclear-encoded photosynthetic gene expression [52]. In this context, the expression of *TRANSLOCON AT THE OUTER MEMBRANE OF CHLOROPLASTS 64-III* (*TOC64-III*) [59], *Ankyrin repeat-containing protein 2* (*AKR2*) [60], *EARLY LIGHT-INDUCABLE PROTEIN* (*ELIP1*) [61], *Tonoplast dicarboxylate transporter* (*TDT*) [62] and VARIEGATED 1 (VAR1) [63] which collectively contribute to chloroplast maturation is subject to IDD4 function (S5B Fig). In addition, our data indicate that IDD4 exerts transcriptional regulation of the blue light photoreceptor PHOTOTROPIN2 (PHOT2) [64] and the J-DOMAIN PROTEIN (JAC1) [65, 66] with assigned functions in the adaptation to light stress (blue light) and photo-morphogenesis in a signal-transduction pathway for photo-chloroplast movement and accumulation (S5B Fig, S4 Table). Altogether, our data indicate that IDD4 is embedded in widely-ramified regulatory pathways thereby exerting transcriptional control on key factors that shape and balance growth and defense.

### IDD4 is a substrate of MPK6

**Fig 5:**
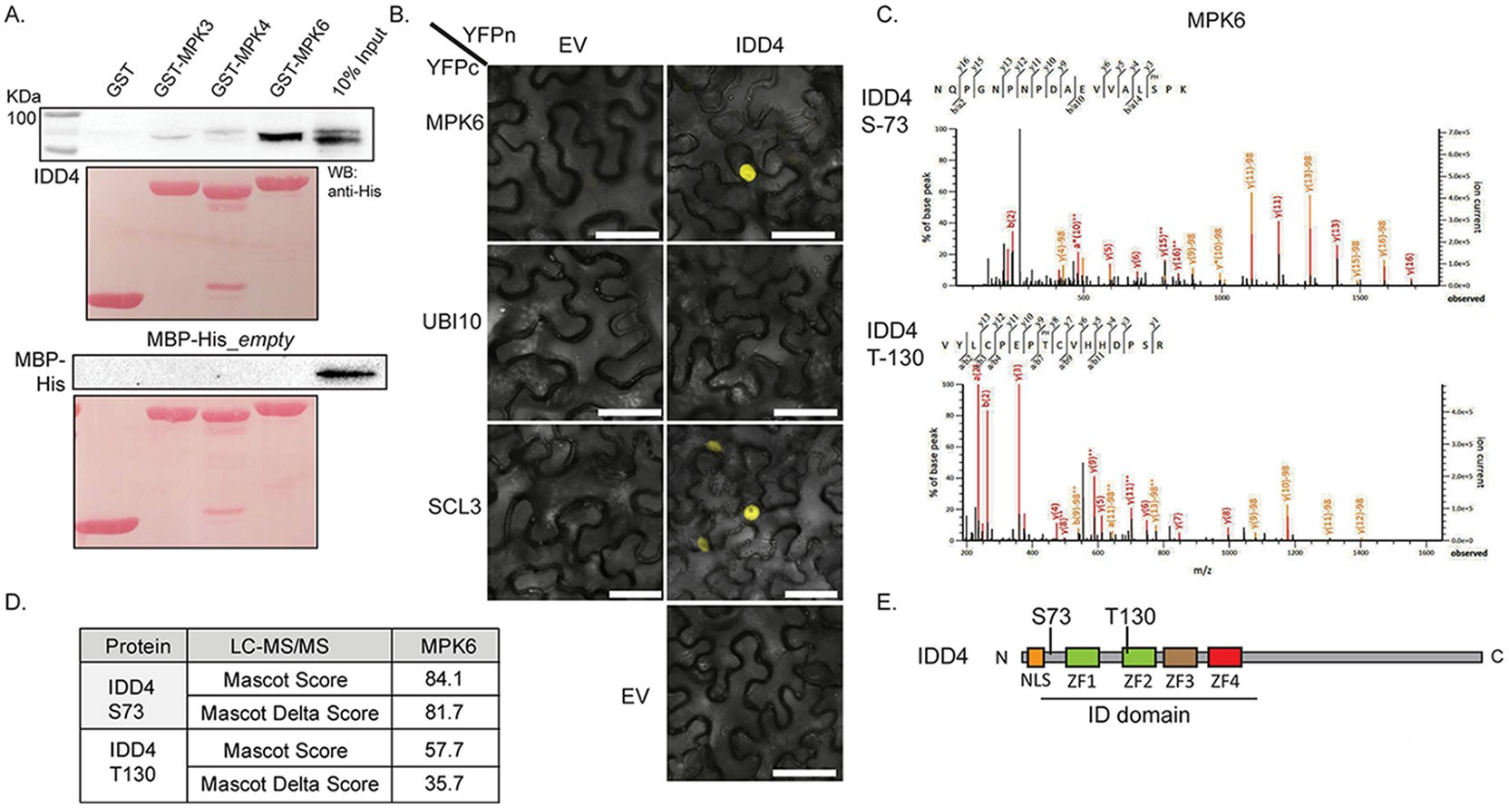
Interaction analysis and kinase assay of IDD4 with MPK6. (**A**) *In-vitro* pull-down assays of MBP-tagged IDD4 showed prevalent interaction with MPK6 and minor association to MPK3 and MPK4. The negative control GST and MBP did not show interaction with MPK3,-4,-6. (**B**) Nuclear interaction study by Bimolecular Fluorescence Complementation of IDD4 with MPK6 in *Nicotiana benthamiana* epidermal cells. SCL3 served as positive control, UBIQUITIN10 and empty vector (EV) acted as negative control. Scale bar = 50 μm. (**C-D**) *in-vitro* kinase assays were performed by using recombinant IDD4 and constitutively active MPK6 and analyzed by LC/MS-MS. Depicted are the spectra of the obtained phosphorylated IDD4 peptides containing SERINE-73 (Ser-73) and THREONINE-130 (Thr-130). (**E**) Domain map of IDD4. IDD4 contains a nuclear-localisation signal (NLS) at the very N-terminus and a highly conserved ID domain that comprises 4 zinc finger (ZF). MPK6-targeted phospho-peptides reside in front of ZF1 (S73) and inside of ZF2 (T130).

To test the interaction between the immune MAPKs MPK3, 4 and 6 with IDD4, we searched for MAPK docking sites in IDD4 that are essential for the binding of substrates to MAPKs. Analysis of the IDD4 amino acid sequence revealed a highly conserved MAPK docking motif that lies between ZF1 and ZF2 (S4A Fig) [67]. The interaction of IDD4 with the immune MAPKs MPK3, MPK4 and MPK6 was tested by *in vitro* pull-down assays using MBP-His-tagged IDD4 and GST-tagged MAPKs (Fig 5A). IDD4 predominately interacted with MPK6. The interaction with MPK3 and MPK4 was at background intensity and the negative controls, single GST and MBP, did not associate with the respective proteins. The association of IDD4 with MPK6 was then evaluated by bifluorescence complementation experiments (BiFC) in *Nicotiana benthaniama*. A strong fluorescence signal was detected in the nucleus of *Nicotiana* epidermis cells that were co-transfected with YFP^N^-IDD4 and YFP^C^-linked MPK6 (Fig 5B). As a positive control, the interaction of SCL3 with IDD4 could be confirmed [16], whereas no association could be detected for UBI10 and as well the empty vector control. The data suggest that among the three immune MAP kinases MPK6 is the principal interaction partner of IDD4.

### MPK6-mediated phosphorylation of IDD4

To evaluate the capability of MPK6 to phosphorylate IDD4, *in vitro* kinase assays followed by LC-MS/MS analysis were performed with a constitutively active version of MPK6 and recombinant IDD4 protein as a substrate. The MS/MS spectra for IDD4 (Fig 5C and 5D) show that MPK6 targets the same amino acid residue (Ser-73) that was identified in several phosphoproteomic studies [43–48]. Moreover, using an inducible MAPKK-activation system, Hoehenwarter et al. (2013) [29] identified the equivalent phosphosite as a putative target of the MAPKs MPK3 and MPK6. Intriguingly, an additional phosphosite in IDD4 (Thr-130) is phosphorylated by MPK6 in the highly conserved N-terminal ID domain (Fig 5E, S4A Fig). Recently, it was shown that MPK6 targets besides the common SP and TP sites PT sites after flg22 treatment [68], suggesting a biological function of Thr-130 after bacterial perception. Notably, the first phosphorylation site resides 11 amino acids upstream of ZF1 whereas the second phosphorylation site is located inside ZF2 (Fig 5E). The post-translational modifications in this region of the ID domain suggest an inherent phosphorylation-dependent regulation mechanism for DNA-binding of IDD4 [17].

### IDD4 binds to the *ID1* motif of the *SAGT1* promoter

**Fig 6:**
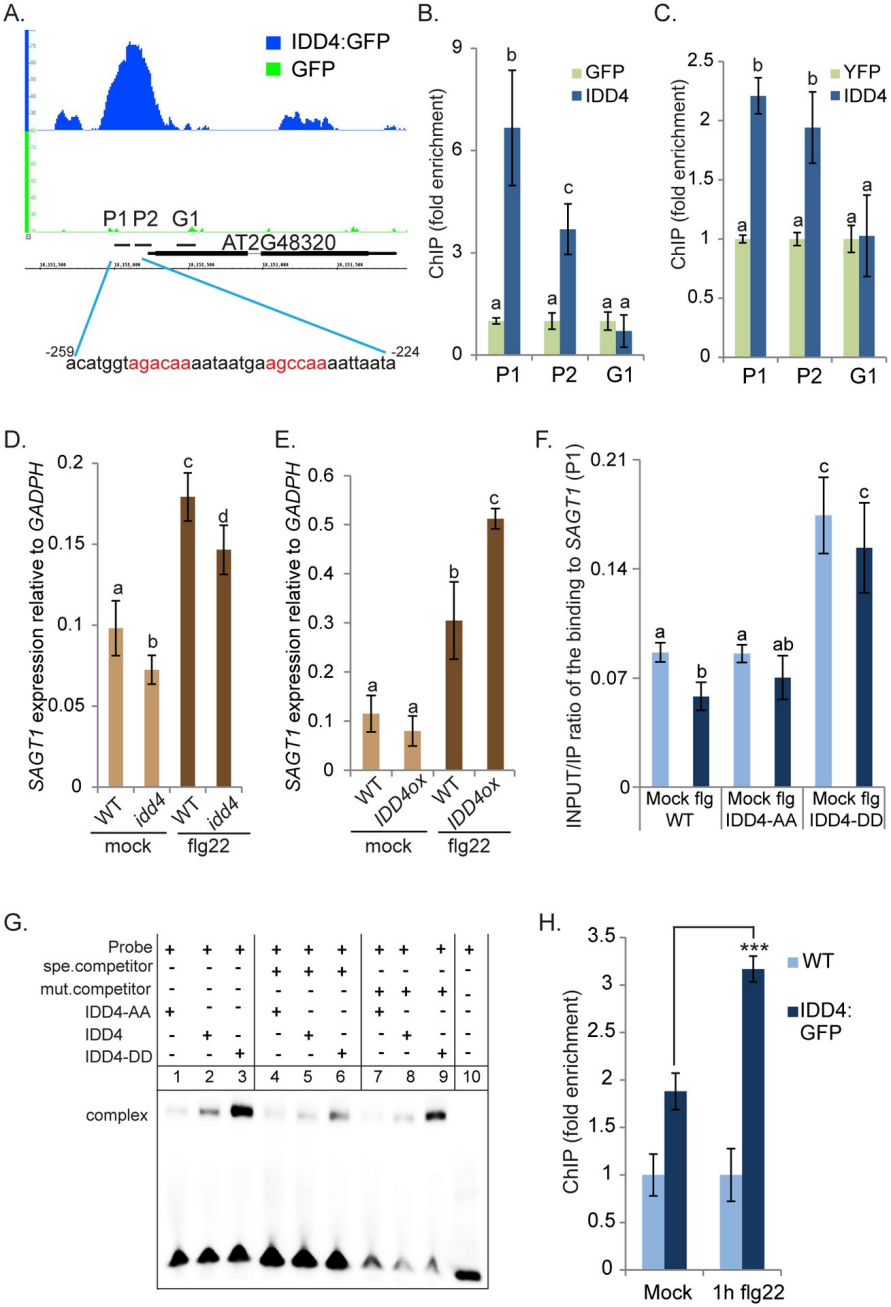
IDD4 associates to the promoter region of *SAGT1* and phospho-modified IDD4 versions show a distinct DNA binding ability. **(A)** Genome Browser snapshots of IDD4 (blue) and single GFP (green) ChIP-SEQ peak on the genomic regions of chromosome 2 [chr2:18,151,900–18,152,300]. Schematic diagram of the *SAGT1* promoter and gene model, upper panel shows the position of the DNA region (*P1*, *P2*, *G1*) used in the ChIP assay and the putative core binding sequences consisting of two *ID1* motifs (red letters) used for EMSA (shown in **Fig. 6G**) are indicated. **(B-C)** ChIP-qPCR by using three biological replicates of *pUBI10*::*IDD4*:*GFP* **(B)** and *pIDD4*::*IDD4*:*YFP* **(C)** expressing plants. Binding of IDD4 to genomic regions close to *SAGT1* was tested with three primer pairs (*P1*, *P2*, *G1*) for each locus. Y-axis shows either the fold enrichment in the *pUBI10*::*IDD4*:*GFP* lines normalized to GFP immunoprecipitation, driven by the *pUBI10* promoter **(B)** or in **(C)** the fold enrichment in the *pIDD4*::*IDD4*:*YFP* lines normalized to YFP immunoprecipitation, driven by the *pIDD4* promoter. **(D-E)** Evaluation of the *SAGT1* expression in *idd4* **(D)** and *IDD4ox lines* **(E)** before and 4 hrs after flg22 treatment compared to WT. The expression of *SAGT1* was reduced under both conditions in the *idd4* mutant **(D**), while it increased in *IDD4ox* lines **(E)** after flg22-application. **(F)** Assessment of the DNA binding activity of IDD4 phospho-modified versions under mock and flg22-treated conditions. Recruitment of IDD4-AA and IDD4-DD to *SAGT1* promoter as determined by ChIP-qPCR. The results are presented as INPUT/IP ratios obtained by signals from ChIP with RFP antibody. Fourteen-day-old seedlings from WT, IDD4-AA:RFP and IDD4-DD:RFP transgenic plants were used for chromatin isolation. ChIP- and input-DNA samples were quantified by qPCR using primer pair *P1*; results shown represent the average of three biological replicates. The protein amount of the different IDD4 variants in transgenic plants is shown by immunoblot assays in S3D Fig. **(G)** Electrophoretic mobility shift assay (EMSA) using truncated IDD4-AA, IDD4-DD and IDD4. Competition experiments were performed using increased amounts (0.5μM, 100x excess) of the indicated unlabeled competitor (spe, specific; mut, mutated). As probe, we used the 35bp sequence inside *SAGT1* promoter that contains two *ID1* motifs, depicted in **Fig 6A (H)** ChIP-based binding study of IDD4 before and after flg22-treatment, represented here by the average of 3 biological replicates. The binding of IDD4 to the *SAGT1* promoter region was increased after 1h of flg22 treatment when compared to untreated samples. IDD4 binding was assessed by using ChIP-qPCR primer *P1* (399a/as). **(B-F, H)** Error bars show ± SEM, statistical significance was analyzed by Student’s test. Asterisks indicate significant differences **p≤ 0.05, **p≤ 0.01, ***p≤ 0.001, letters above bars represent significant groups *p*≤0.05.

The elevated resistance of the *idd4* mutant accompanied by the elevated expression of genes involved in salicylic acid (SA) metabolism and signaling suggest a function of IDD4 in these processes. The 500 bp upstream region of the translational start sequence (TSS) of the *SALICYLIC ACID GLUCOSYLTRANSFERASE 1* (*SAGT1*) gene has two *ID1* motifs in the promoter region between positions -259 and -224 upstream of the TSS (Fig 6A) and becomes bound by IDD4 with a log_2_(FC) of 2.20. This finding was confirmed *in vivo* by ChIP_qPCR using transgenic lines expressing IDD4-GFP under the *UBI10* promoter (Fig 6B) and its native promoter (Fig 6C) showing consistently that IDD4 binds to the *SAGT1* promoter region P1 and P2 (-300 to 0) close to the TSS. *SAGT1* converts SA to the biologically inactive storage forms SA-2-l-β-D-glucoside (SAG) and SA-glucose-ester (SGE) [69]. In agreement with this function, transgenic *SAGT1ox* plants exhibited increased susceptibility to *Pst DC3000* and showed reduced levels of free SA [70, 71].

### IDD4 regulates expression of *SAGT1*

The expression of *SAGT1* is induced by *Pst DC3000* infection and Methyl-SA [69, 71]. Genome-wide DNA binding and ChIP-qPCR analysis showed that IDD4 binds to the *SAGT1* promoter and consequently identifies *SAGT1* as a direct target of IDD4. Indeed, *SAGT1* transcript levels are reduced in the *idd4* mutant (log_2_(FC) -0.39, *p*<0.01) in untreated conditions and after flg22 treatment (log_2_(FC) -0.4, *p*<0.05) (S2 Table) when compared to WT plants. These results could be confirmed by qPCR (Fig 6D). On the other hand, *SAGT1* transcript levels are enhanced in *IDD4ox* (Fig 6E) after flg22-treatment, indicating that IDD4 acts as a positive regulator of *SAGT1* expression.

### IDD4 phosphorylation determines *ID1*-motif DNA-binding

To study the biological function of IDD4 phosphorylation *in planta*, we replaced Ser-73 and Thr-130 by aspartic acid (D) to generate a phospho-mimicking IDD4 version S-73-D, T-130-D (DD) or by alanine (A) to produce a phospho-dead IDD4 S-73-A, T-130-A (AA) version. As shown in S4E and S4F Fig, both phospho-modified IDD4 versions still interact with the M5GAI (RGA/DELLA) protein in Y2H and SCL3 in BiFC analysis. These results suggest that phosphorylation of IDD4 in the first 2 ZFs does not hamper the interaction with these partners. To test the influence of phosphorylation on the DNA binding capability of IDD4, we produced stable transgenic plants expressing the phospho-modified IDD4:RFP versions controlled by the *UBIQUITIN10* promoter. For further studies, transgenic lines were selected that exhibit comparable transcript levels of IDD4-modified-versions *IDD4-AA* and *IDD4-DD* thereby accumulating equal amounts of the encoded protein (S3D Fig). The phenotype of the IDD4 phospho-modified plants is indistinguishable from WT and the shoot fresh weight of 18 day-old *IDD4-AA* lines is slightly reduced compared to *IDD4-DD* lines but insignificant to WT. The root fresh weight of *IDD4-AA* and *IDD4-DD* lines is comparable to WT (S2G–S2I Fig). These lines were used for ChIP-PCR to determine binding preferences of phosphorylated IDD4. Quantitative PCR of DNA that was immunoprecipitated by RFP antibody confirmed that the phospho-mimicking version IDD4-DD shows higher binding affinity to the *SAGT1* promoter compared to the IDD4-AA line or WT control under both flg22 and mock-treated conditions (Fig 6F). The inability of IDD4-AA to increase DNA binding to the *SAGT1* promoter after flg22-treatment demonstrates the importance of the flg22-mediated post-translational modification of IDD4 at the phosphorylation sites. In order to analyze whether the binding ability enhancement of the unmodified IDD4 to the *ID1* element also occurs *in vivo* in the context of PAMP signaling, ChIP of IDD4:GFP lines was performed 1 hour after flg22 treatment (Fig 6H). Previously, we tested whether flg22 perception influences IDD4 protein stability by analyzing *IDD4ox* lines for IDD4:GFP degradation at 30 and 60 min after flg22 treatment in 10 day-old seedlings. No change of stability was seen under these conditions by Western blotting or fluorescence microscopy in roots (S3E and S3F Fig) demonstrating that flg22-perception does not compromise IDD4 protein stability. However, the association of IDD4:GFP to the *SAGT1* promoter in the ChIP approach was significantly elevated after flg22 application unlike the IDD4:GFP mock-treated control (Fig 6H). This finding suggests that PAMP-treatment determines the binding ability of IDD4 to the *ID1* motifs of the *SAGT1* promoter.

These studies were extended by DNA-shift experiments using the recombinant N-terminal domain of IDD4 (IDD4-WT, IDD4-AA and IDD4-DD), containing the 2 phosphorylation sites and the 4 zinc-finger-containing ID domain previously shown to be necessary for DNA binding and interaction with other partners, respectively. As a probe, the sequence of the *SAGT1* promoter region -259 to -224 (Fig 6A) was used that harbours 2 *ID1* motifs and was shown to be targeted by IDD4:GFP in the ChIP-SEQ and ChIP-qPCR approaches (Fig 6A–6C). Predominant binding of IDD4-DD to the biotinylated *ID1* motifs could be confirmed; whereas IDD4-AA and unmodified IDD4 exhibited much weaker binding preferences (Fig 6G). By adding an unlabeled specific competitor to the reaction, the interaction between IDD4-DD and the *ID1* probe could be significantly diminished. By contrast, the addition of an unlabeled probe containing mutated *ID1* motifs only resulted in a minor competition on IDD4-DD binding. Taking altogether, these results demonstrate the enhanced DNA-binding ability of the phospho-mimiking IDD4 version.

### Phospho-modified IDD4 versions show opposite transcriptional activity *in vivo*

**Fig 7:**
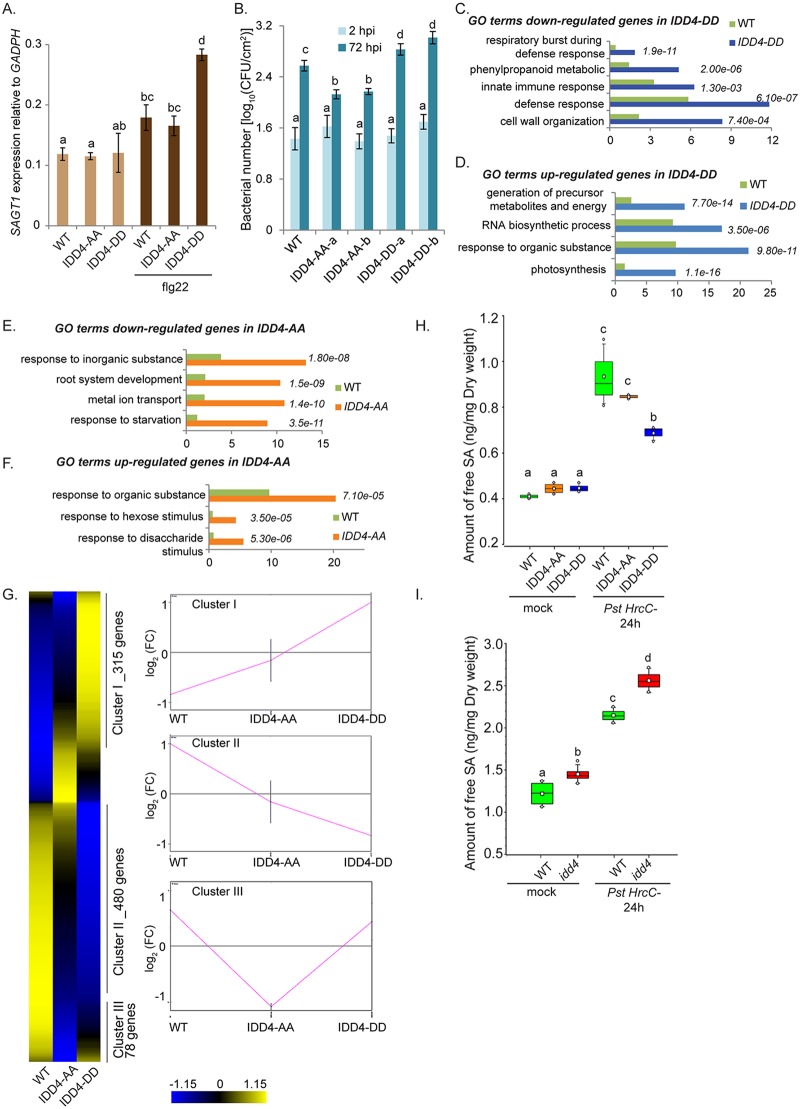
Phospho-modified IDD4 versions exert a distinct transcriptional activity, SA accumulation and susceptibility against *PstDC3000 hrcC-* infection. **(A)** Phospho-modified IDD4 lines show a distinct *SAGT1* expression 4 hrs after flg22 treatment. **(B)** *Pst DC3000 hrcC-* infection levels in two independent *IDD4-AA* and *IDD4-DD* lines. Plants of three biological replicates (*n* = 30) were spray-inoculated with a bacterial suspension at OD_600_ 0.2. Density of colony forming units (cfu) was analyzed 2 and 72 hours post inoculation (hpi). **(C-D)** GO term analysis of down-regulated **(C)** and up-regulated genes **(D)** in the *IDD4-DD* line (*p*≤0.05). **(E-F)** GO term analysis of down-regulated **(E)** and up-regulated genes **(F)** in the *IDD4-AA* line (*p*≤0.05). GO analysis was performed by using AgriGo (TAIR10). **(G)** Transcriptome compositions of *IDD4-AA* and *IDD4-DD* lines with respect to significant differentially regulated genes (*p*<0.05). Three main clusters generated illustrate a distinct and partly opposite expression of differentially expressed genes in the particular genotypes. **(H-I)** Quantitative analysis of free SA levels in WT, *IDD4-AA* and *IDD4-DD* lines **(H)** and as well as in WT and *idd4* mutant plants **(I)** analysed by LC-MS/MS, before and 24hrs after *Pst DC3000 hrcC-* infection. Plants of three biological replicates were spray-inoculated with a bacterial suspension at OD_600_ 0.2. Boxes represent the 25th and 75th percentiles and the inner rectangle highlight the median, whiskers show the SEM, and outliers are depicted by dots (Min/Max range). **(A, B, H, I)** Error bars show ± SEM, statistical significance was analyzed by Student’s test, letters above bars represent significance groups, *p*≤0.05.

After obtaining indications that the DNA-binding property of IDD4 might depend on the phosphorylation status *in vivo* and *in vitro*, we analyzed whether the phosphorylation status of IDD4 determines the transcriptional activity as well. To prove the biological relevance of the transcriptional activity of IDD4, the expression of *SAGT1* was studied in the phospho-modified versions expressing lines under untreated conditions and after flg22 application (after 4 hrs). The relative gene induction level of *SAGT1* after flg22 treatment was significantly induced in the *IDD4-DD* line, reminiscent to the *IDD4ox* line (Fig 6E), however, unlike to the *IDD4-AA* line that does not show an increased expression level when compared to WT (Fig 7A). These findings suggest that IDD4-DD acts as an activator of *SAGT1* gene expression. By contrast, the expression of *SAGT1* in the phospho-modified lines under untreated conditions was indistinguishable from WT, accordingly to the WT-like expression of *SAGT1* in the *IDD4ox* line (Fig 6E). Altogether, these results imply the importance of the IDD4 phosphorylation status to initiate target gene expression in a flg22-dependent manner.

### Phospho-modified IDD4 versions show different PTI responses and transcriptional composition

After showing that phosphorylation of IDD4 coordinates gene expression, the biological relevance of this mechanism was assessed after hemi-biotrophic pathogen infection. Therefore, we measured *Pst DC3000 hrcC-* infection levels in two independent transgenic lines harbouring *IDD4-AA* and *IDD4-DD*. At 2 hrs after spray-infection of the different phospho-modified line, similar bacterial cfu were obtained, indicating that the accessibility of the bacteria to the leaf apoplast tissue was not affected. However, 72 hrs after plant infection, the proliferation levels of the bacteria were significantly elevated in both *IDD4-DD* lines compared to *IDD4-AA* lines and WT. Furthermore, the bacterial titers in the *IDD4-AA* lines were reduced even below WT levels indicating that the posttranslational-modification of IDD4 impacts bacterial growth and defense response (Fig 7B). In order to analyse the susceptibility of the modified IDD4 expressing lines at the molecular level, the transcriptomes of 14 day-old seedling were studied by *RNA*-Hiseq of 3 biological replicates. The transcriptome composition of *IDD4-DD* lines revealed 429 genes as being down-regulated and 354 up-regulated (*p*<0.05) when compared to WT or *IDD4-AA* lines. GO terms of down-regulated genes could be determined for defense response, respiratory burst and innate immunity (Fig 7C, S6 Table). GO terms of the up-regulated genes in *IDD4-DD* highlighted gene functions in energy metabolism, RNA biosynthesis process and photosynthesis (Fig 7D). On the other hand, GO terms of down-regulated genes in *IDD4-AA* (*p*≤0.05, 211 genes) referred to response to starvation, root system development and metal ion transport (Fig 7E). Furthermore, GO terms for up-regulated genes in *IDD4-AA* (p≤0.05, 169 genes) emphasize gene functions for sugar and carbohydrate response (Fig 7F, S6 Table). In summary, the GO term analysis indicates a distinct molecular setting between *IDD4-AA* and *IDD4-DD* lines, e.g. favouring growth and energy metabolism at the expense of defense response in the case of the phospho-mimicking (DD) line. The transcriptome compositions of *IDD4-AA* and *IDD4-DD* lines with respect to significant differentially regulated genes (*p*<0.05) resulted in 3 main clusters illustrating a distinct and partly opposite expression of DEGs in the particular genotypes (Fig 7G). Cluster I and II show a profound differential regulation of downstream targets in the *IDD4-DD* lines whereas their deregulation in *IDD4-AA* lines is rather moderate and oppositely to the *IDD4-DD* line, such as in cluster I. To assess the biological functions of DEGs in the respective clusters, we carried out a GO term analysis. Cluster I highlights among others GO terms for response to water deprivation (5.4e^-13^) and abiotic stress stimulus (2.1e^-08^), whereas genes in cluster II were dedicated to the GO terms plant-type cell wall organisation (6.6e^-10^) and defense response (1.7^e-07^). By contrast, cluster III emphasises genes that are down-regulated in the *IDD4-AA* lines and moderately up-regulated in the *IDD4-DD* lines supporting additionally the notion of a distinct regulation of subsets of target genes indicating a distinct transcriptome composition (Fig 7G). In this context, GO terms for cluster III could be assigned to gene function in response to starvation (4.6e^-08^) and inorganic cation transport (3.1e^-07^). The molecular characterisation by qPCR of the phospho-modified lines 4 hours after flg22 treatment showed differential regulation of the SA response marker *PR1* being higher expressed in *IDD4-AA* lines than in *IDD4-DD* and WT (S4B Fig). By contrast, the expression of the SA-signaling inhibitors *WRKY38* [72] and *NIMIN1* [73] are elevated in the *IDD4-DD* lines and the expression of *NIMIN1* is reduced in *IDD4-AA* compared to WT and *IDD4-DD* line (S4C and S4D Fig). These expression patterns of SA-signaling markers indicate a reduced SA response in the *IDD4-DD* lines. Therefore, to determine the concentration of free SA in the IDD4-phospho-modified lines after *Pst DC3000 hrcC-* infection, hormonal measurements were conducted. Quantitative analysis of free SA in 3 biological replicates of WT, *IDD4-AA* and *IDD4-DD* transgenic lines before pathogen infection (Fig 7H) revealed comparable amounts of about 0.42 ng/mg dry weight. However, 24 hrs after being challenged by *Pst DC3000 hrcC-*, WT and *IDD4-AA* lines showed strongly increased comparable levels of free SA, whereas the *IDD4-DD* lines exhibited significantly lower amounts. These findings are compatible with the notion that the higher susceptibility of the *IDD4-DD* lines to *Pst DC3000 hrcC-* is to some extent caused by its diminished SA levels as reflected by the elevated expression of the negative SA regulators *NIMIN1*, *WRKY38* and *SAGT1*. If IDD4 and in particular its phosphorylation status contributes to SA homeostasis, then the SA accumulation after pathogen treatment should as well as be compromised in the *idd4* mutant. Therefore, eventually, we determined the free SA levels in the *idd4* mutant 24 hrs after *Pst hrcC-* infection. Interestingly, we found already a moderately elevated SA level in the *idd4* mutant (Fig 7I). Furthermore, the pathogen-induced SA rise was also higher than in infected WT plants. Taking into account the higher resistance of *idd4* against hemi-biotrophic pathogens (Fig 1I and 1J) and the differential regulation of components contributing to SA metabolism/ catabolism such as *SAGT1* (Fig 6D and S2F Fig), then these results corresponds with a function of IDD4 in the modulation of SA homeostasis. All in all, these findings suggest a profound biological significance of IDD4 phosphorylation to coordinating plant defense with growth and developmental processes.

## Discussion

This work illustrates the involvement of the *IDD* family member 4 in the regulation of defense responses and in processes governing plant growth. IDD4 is embedded in widely-ramified regulatory pathways and exerts transcriptional control on key factors that shape and balance growth with defense.

### IDD4 contributes to distinct functional networks that coordinate growth and defense

Plant response to pathogen attack often requires “trade-off” processes in which resources initially dedicated to growth and pattern formation will be redistributed to pay the high metabolic costs of defence. It follows that plants have to precisely regulate resources to be allocated to fight against a pathogen [74]. In this regard, it was shown that the members of the PHYTOCHROME-INTERACTING FACTORS (PIFs) play a pivotal role in the regulation of growth defense trade-offs to adapt to changing environmental conditions [75]. PIFs redundantly inhibit skotomorphogenesis and individually regulate other light-mediated processes such as shade avoidance responses, chloroplast differentiation and seed germination but their own transcriptional regulation is poorly understood [76]. In addition to the light responses, some PIF members including PIF4 are involved in the hormonal responses (gibberellic acid (GA), brassinosteroid (BR), and auxin) [53]. In this regard, PIF4-mediated thermosensory growth and architecture adaptations are directly linked to suppression of immunity at elevated temperature. Accordingly, the *pif4-101* and *pifQ* (*pif1*, *pif3*, *pif4*, *pif5*) quadruple mutant exhibited increased resistance to *PstDC3000*, demonstrating PIF4 as a positive regulator of growth and development and negative regulator of immunity [77]. Interestingly, we found that IDD4 binds to the promoter region of several PIF members (*PIL1*, *PIL2/PIF6*, *PIL5/PIF1*, *PIF3*) and photoreceptor PHYTOCHROME B (PHYB) that regulates PIF protein levels through promoting light-dependent protein degradation [75] thereby supporting resistance against *PstDC3000* [77]. The association of IDD4 to these promoter regions shows an important function in the transcriptional regulation of these system integrators in plant development. In addition, our ChIP-SEQ analysis revealed the association of IDD4 to the G-Box, also called PIF4-box (Fig 3B), which is highly enriched in PIF4 target promoters previously shown by PIF4 ChIP-SEQ analysis [78]. The PIF4 DNA binding ability depends on its capacity to form hetero- and homodimers. The shared usage of the G-Box *cis*-regulatory elements by PIF4 and IDD4 might be mediated by heterodimer formation of these factors to shape the DNA-binding ability and expression of particular gene subsets. We have shown that IDD4, in accordance with PIF4, acts as repressor of immunity against hemi-biotropic pathogens. However, the enhanced growth of the *idd4* mutant demonstrates that IDD4 acts as a suppressor of plant growth and development, unlike PIF4 that positively regulates these processes. Consequently, we suspect that IDD4 and PIF4 cooperatively mediate the expression of defense-related genes that might antagonistically contribute to processes promoting growth and development. Moreover, IDD4 was previously reported being transcriptionally repressed by *KAN* and *REV*, and *IDD4ox* lines are compromised in leaf blade formation and leaf size [18]. The overexpression of IDD4 causes downward leaf curling, resembling reduced HD-ZIPIII gene function which indicates a negative feedback regulatory network of the three factors. The discovered binding of IDD4 to promoter regions of *KAN* and *REV* suggest an up to now uncharacterized feedback regulation. In addition, pathogen attack has been shown to suppress components of photosynthesis at the level of gene expression and protein abundance; and defense exerts a negative impact on photosynthesis which results in a reduction of components essential for light harvesting and carbon fixation [74]. We show that IDD4 affects the expression of genes involved in chloroplast maturation, localization in response to changing light conditions and photo-morphogenesis. Furthermore, the auto-immune phenotype of *idd4*, reflected by the internal increase of H_2_O_2_, indicates a role of IDD4 in the photo-respiratory H_2_O_2_ accumulation.

### Indications for flg22-mediated phosphorylation of IDD4

We showed that IDD4 is phosphorylated by the immune MAPK MPK6 whose activation is triggered *in planta* upon PAMP-perception to modulate immune reaction [33]. We found that the IDD4 phospho-modified versions behave differently regarding the DNA-binding ability, target-gene activation and response to pathogen-attack. In this context, the IDD4-phospho-mimiking version (IDD4-DD) shows stronger recruitment to the DNA and acts as transcriptional activator of *SAGT1* expression. By contrast, the phospho-dead version (IDD4-AA) displays weak DNA-binding and low transcriptional activation after flg22-treatment. By exploiting the native IDD4 version, we found that IDD4-binding and transcriptional activity are increased upon PAMP-perception. After flg22-application, the recruitment of IDD4 to the DNA and the transcriptional activity are elevated in accordance with the results of the phospho-mimicking IDD4-DD version. Moreover, the opposite behavior of IDD4-AA and its unresponsiveness to flg22-treatment support the notion of a post-translational modification-based mechanism to regulate IDD4 DNA-binding properties. Phosphosite-dependent transcriptional deactivation of IDD8 mediated by AKIN 10 in the process of carbon metabolism was recently reported [79]. The phosphorylation of IDD8 at Ser-178 and Ser-182, which reside both in the fourth ZF domain did not affect the subcellular localization and DNA-binding property of IDD8 but diminished the transcriptional activation activity. Interestingly, the function of a putative transactivation domain might be compromised by the phosphorylation of the closely adjacent ZF4 [45]. Recently, the importance of ZF4 for protein-protein interaction of IDD10 and IDD3 with the SCR-SHR complex was reported [17]. Furthermore, the transcriptional activity of IDD10 is modulated by reciprocal interactions with IDD3, SCR and SHR [80]. Therefore, it is conceivable that the phosphorylation of ZF4 in IDD8 interferes with the association of coactivators. In summary, the post-translational modification of particular ZFs in IDD4 and IDD8, as the discussed phosphorylation events, change their characteristic traits and can be considered as a general IDD/BIRD-regulation mechanism to modulate DNA-binding ability, protein-protein interaction and transcriptional activity.

### IDD4 and DELLA/RGA might synergistically modulate immunity

Recently, the gibberellin (GA)-inhibitory DELLAs were introduced to control plant immune responses by modulating the balance of JA and SA [81]. Notably, DELLAS cannot directly bind to the DNA because of the lack of a DNA interaction—domain. However, they act as co-activators or repressors, respectively, by binding to transcription factors in a stress-dependent manner in order to coordinate target gene expression. We showed the interaction of IDD4 with the DELLA/GAI. The IDD family members 2, 3, 4, 5, 9 and 10 serve as transcriptional scaffolds to enable transactivation activity of the DELLA/RGA of the GRAS-family [16] [22]. *In vitro* studies have confirmed the transcriptional activation of DNA-bound IDDs upon the association of RGA and the subsequent expression of the *SCL3* locus [16]. Interestingly, genome-wide DNA-binding studies on DELLA/RGA revealed the enrichment of the *ID1 cis*-motif in about 28% of target sequences underpinned by a total *p* value of 2.3e^-10^. The occurrence of the *ID1* motif indicates a common set of RGA and IDD4 controlled genes [82]. In this context, we identified a substantial overlap of 20.4% of RGA and IDD4 target genes and found associated GO terms, among others, for response to salicylic acid stimulus, light stimulus and regulation of defense response (S3 Table). Interestingly, the introduced *quadruple*-*DELLA* mutant is more resistant to *Pst DC3000* compared to WT and accumulates higher levels of free SA after pathogen attack [81], demonstrating that DELLAs promote disease susceptibility to hemi-biotrophic pathogens and repress the SA-defense pathway. Therefore, the reduced susceptibility to bacterial infection in the association with the elevated SA levels of the *idd4* mutant, and the reduced SA accumulation in the *IDD4-DD* lines after *Pst HrcC-* infection suggest a synergistic interaction of IDD4 and DELLA/RGA proteins in the regulation of selected defense processes. Moreover, GAI-ASSOCIATED FACTOR1/IDD2 is involved in the regulation of GA homeostasis and signaling in *Arabidopsis* for binding to genes which are part of GA feedback regulation. GA converts the IDD2 complex consisting of DELLA, IDD2 and TOPLESS RELATED from a transcriptional activator to a repressor upon the degradation of DELLA [22]. Similarly, the co-repressor SCL3 interacts competitively with DELLAs for the binding to IDD proteins to antagonistically regulate downstream gene expression to control GA signaling pathways [21].

### IDD4 specificity to *ID1* motif binding

To assess the distribution and abundance of the core *ID1* motif (AGACAA), we performed a genome-wide *in silico* search of the 500 bp upstream regions in the promoter regions of IDD4-bound genes for the occurrence of at least two *ID1*-core motifs. Previous publications suggested the *ID1-cis* motif as the main binding sequence of different IDD family members, including IDD4 [22] [44] and it was shown that IDD4 binds to the *ID1*-core sequence inside the *SCL3* promoter *in vitro* [16]. Noteworthy, our *in vivo* ChIP-SEQ data confirm the binding of IDD4 to the upstream regulatory sequence of *SCL3* (S3B Fig, S3 Table). Our analysis revealed 2840 genes harbouring this element up to 8 times whereas 82.9% of the genes contained two *ID1*-core motifs (S5 Table). This high number of genes with two *ID1-core* motifs in their promoter regions is surprising and raises the question of the specificity of IDD4 DNA-binding and how target gene activation can be achieved. We provide evidence that phosphorylation of IDD4 is a post-translational regulatory mechanism to trigger IDD4 DNA-binding. In order to coordinate the binding of IDD4 to the *ID1* motif, binding partners might be involved in a developmental- and stress-dependent manner modulating the accessibility of the binding sites to IDD4. For example, IDD4 activity could be orchestrated by the formation of IDD4 homo- and heterodimers with other transcriptional regulators in a developmental or stress-dependent manner. Further mechanisms might include different splice variants as shown for IDD14, which are generated by cold stress to form a competitive inhibitor regulating starch metabolism. IDD14β lacks one functional DNA binding domain but is still able to create heterodimers with the functional IDD14 form (IDD14α). However, IDD14α–IDD14β heterodimers have diminished DNA-binding activity to their target promoter [83]. Slight modifications in the ratio of functional transcription factors and alternatively spliced variants could sustainably affect the expression of target genes. In this context, it is worth mentioning that IDD4 also forms a second splice variant (IDD4.2) (TAIR-database), which contains the 4 zinc finger-containing ID domain, but excludes the phosphosite Ser-73. This means that IDD4 can presumably form heterodimers consisting of IDD4.1-IDD4.2 as well as homodimers with each of the two splice variants. It is well conceivable that the IDD4.2 homodimer without the phosphorylation site could act as a competitive inhibitor of the phosphorylated IDD4.1 protein and further research is necessary to pursue these possibilities.

## Materials and methods

### Plant material and growth conditions

Experiments were performed by using *Arabidopsis thaliana* of the Columbia accession grown on soil in plant growth chambers (Percival Scientific) under short-day conditions (8h light/ 16 h dark) at 22°C. *Nicotiana benthamiana* were grown under long day conditions (16 h light + 8 h darkness) at 28 °C. *idd4* (Salk_148352C) seeds were obtained from NASC.

### RNA Extraction and real-time quantitative PCR analysis

Total RNAs were extracted from 14 day-old seedlings, grown on sugar-free Murashige and Skoog (MS) plates under long-day conditions. We used the NucleoSpin RNA Plant (MACHEREY-NAGEL) kit, according to the manufacturer’s instructions. First strand cDNA was synthesised from 5μg of total RNAs using SuperScript First-Strand Synthesis System for RT-PCR according to the manufacturer’s instructions. The cDNA stock was diluted to a final concentration of 25ng/ul. Subsequently, 500nM of each primer was applied and mixed with LightCycler 480 Sybr Green I Master mix for quantitative PCR analysis, according to the manufacturer’s instructions. Products were amplified and fluorescent signals acquired with a LightCycler 480 detection system. The specificity of amplification products was determined by melting curves. GADPH was used as internal control for signals normalisation. Exor4 relative quantification software automatically calculates relative expression level of the selected genes with algorithms based on ΔΔCt method. Data were from duplicates of at least three biological replicates. All the sequences of primers used are given in S7 Table.

### Immunoblot and ChIP Analyses

Nuclear proteins were extracted from 14 day-old on half MS-grown seedlings. After quantification with the Bradford method, equal amounts of proteins were resolved by SDS-PAGE and then transferred to a polyvinylidene difluoride membrane using a Mini-Protean 3 Cell. Immunoblot analysis was performed using 1 μg/mL primary polyclonal antibodies raised against GFP and then with secondary antibodies conjugated to alkaline phosphatase. Antibody complexes were detected by chemiluminescence using the Immun-Start AP Substrate kit. ChIP assays were performed on 14 day-old in half MS-grown seedlings using Anti-GFP antibody—ChIP Grade and RNA polymerase II antibodies. Briefly, after plant material fixation in 1% (v/v) formaldehyde, the tissues were homogenised, and the nuclei were isolated and lysed. Cross-linked chromatin was sonicated using a water bath Bioruptor (Diagenode; 15-s on/15-s off pulses, 15 times). The complexes were immunoprecipitated with antibodies overnight at 4°C with gentle shaking and incubated for 1 h at 4°C with 50 μL of Dynabeads Protein A. Immunoprecipitated DNA was then recovered and analysed by qRT-PCR. An aliquot of untreated sonicated chromatin was processed in parallel and used as the total input DNA control.

### Bioinformatics analysis of ChIP-SEQ data

Sequencing of Col-0, IDD4:GFP sample material was performed in Illumina Hi-Seq2000 platform. About 47 million and 53 million paired-end reads (insert size of 300 bp) with 125 bp read length were obtained for Col-0 and IDD4 respectively. Quality statistics of reads were analyzed using FASTQC [http://www.bioinformatics.babraham.ac.uk/projects/fastqc/]. Trimming and filtering of reads were performed using trimmomatic [84]. Parameters for read quality filtering were set as follows: Minimum length of 36 bp; Mean Phred quality score greater than 30; Leading and trailing bases removal with base quality below 3; Sliding window of 4:15. Filtered reads were aligned to TAIR10 using Bowtie (Unique mapping of reads was adopted) and enriched regions were identified using MACS2 [85]. Parameters for peaks detection were set as follows: Number of duplicate reads at a location: 1; Bandwidth: 300; mfold of 5:30; q-value cutoff: 0.05. Peaks were identified against input DNA sequence. Results from all three peak callers (MACS2, SISSR, HOMER) have shown very high correlation. Bedtools was used for manipulation of these genomic peak intervals [86]. Identification of putative IDD4 binding motifs (p-value < 0.05) at called peak positions was done using HOMER [87]. Coverage and enrichment of functional elements from TSS to TES and their flanking region were visualized using NGSplot [88]. The complete ChIP-SEQ data sets are available at the GEO repository (GEO accession GSE120068).

### Bioinformatics analysis of RNAseq data

Sequencing was performed on each library to generate 101-bp paired-end reads on Illumina HiSeq2500 Genome Analyzer platform. Read quality was checked using FastQC and low quality reads were trimmed using the Trimmomatic version 0.32 (http://www.usadellab.org/cms/?page=trimmomatic) with the following parameters: Minimum length of 36 bp; Mean Phred quality score higher than 30; Leading and trailing bases removal with base quality below 3; Sliding window of 4:15. After pre-processing the Illumina reads, the transcript structures were reconstructed using a series of programs, namely, TopHat (ver. 2.1.1; http://tophat.cbcb.umd.edu/) for aligning with the genome, and Cufflinks (ver. 2.2.1; http://cufflinks.cbcb.umd.edu/) for gene structure predictions. For TopHat, the Reference-*Arabidopsis thaliana* (TAIR10) genome (https://www.arabidopsis.org) was used as the reference sequences with a maximum number of mismatches as 2. To identify the differentially expressed genes, the following parameters were used: p-value of 0.05 with a statistical correction using Benjamini Hochberg FDR of 0.05 in cuffdiff. A cut-off of 2 fold up- or down-regulation has been chosen to define the differential expression. After processing the data, visualisation of differential expression was done using cummeRbund v2.14.0 (http://bioconductor.org/packages/release/bioc/html/cummeRbund.html). Differentially regulated genes that are common among the samples were identified using Venny. The complete *RNA*seq data is available at the GEO repository (GEO accession GSE120068).

### Bioinformatic analyses/ GO term analysis

For the GO term analysis, AGRIGO analysis tool was used (http://bioinfo.cau.edu.cn/agriGO/, [30] by using significantly differentially expressed genes between the tested conditions. Protein-network analysis was performed by using STRING [46]. Putative MAPK docking site of IDD4 was searched using the ELM program (http://elm.eu.org/).

### Plasmid construction

Whole seedling Arabidopsis cDNA library was used to amplify the coding sequence (cds) of the *IDD4* and *SCL3*. Subsequently, the entry clone was generated by introducing the cds either in the *pENTR*- or *pCR8/GW/TOPO*-vector. Subsequently, entry clones were used to generate protein expression constructs (*pDEST-MBP* [89]) and protein localisation vectors, fused to GFP/RFP driven by the Ubiquitin promoter [90]. Site-direct mutagenesis of *IDD4* was performed in a 2 step approach. First oligo-nucleotide based introducing of the base pair exchange results in two truncated cDNA fragments with an overlapping region. Secondly, both fragments were fused and amplified by using oligos that bind in the end and beginning of the total cDNA. GAI (amino acids 148 to 533), RGA, IDD4, IDD4-AA, IDD4-DD cDNA inserted into *pDONR207* by Gateway cloning were recombined with *pGADT7* (AD) or *pGBKT7* (BD) to generate BD-GAI, AD-IDD4, AD-IDD4-AA and AD-IDD4-DD. All the sequences of primers used are given in S7 Table.

### Yeast two-hybrid assays

Direct interaction assays in yeast were carried out following the small-scale LiAc yeast transformation procedure. The N-terminal part of the DELLA proteins is subject to autoactivation in yeast two-hybrid assays; therefore, only the C-terminal domain of GAI (amino acids 148 to 533) was recombined by Gateway with *pGBKT7* to generate BD-GAI. Yeast strain AH109 was co-transformed with BD-GAI and AD-IDD4/IDD4-AA/IDD4-DD or empty vector (*pGADT7*), and interaction tests were surveyed on selective media lacking Leu, Trp, Ade and His.

### Quantification of SA

Plant materials were lyophilised and ground in a bead beater. Aliquots (about 5 mg dry weight) of powdered tissues were extracted with 400 μL of 10% methanol containing 1% acetic acid and internal standards (11.1 ng of 2H_4_-SA). The samples were extracted in the bead beater for 1 min, placed in ice for 30 min, and then centrifuged at 13,000 g for 10 min at 4°C. The supernatant was carefully removed and the pellet re-extracted with 400 μL of 10% methanol containing 1% acetic acid. Following further 30 min incubation in ice, the extracts were centrifuged and the supernatants combined. The samples were filtered through 0.22 μm PTFE filters before LC-MS/MS analysis. Analysis SA was performed by comparing retention times and mass transitions with the standards using an Agilent 1200 HPLC coupled to an Q-TRAP 5500 MS with an electrospray source. Chromatographic separation was carried out at 35°C on a Phenomenex Gemini C18 (150×2.0 mm, 5 μm) column with the solvent system formic acid/acetonitrile/water (0.1/94.9/5, v/v/v; mobile phase A) and formic acid/ acetonitrile/water (0.1/5/94.9, v/v/v; mobile phase B). The gradient used was 0–20 min, 0%-100% A; 20–25 min, 100% A; 25–26 min, 100%-0% A; 26–36 min, 0% A. To reduce contamination of the MS, the first 5 min of the run was directed to waste using the inbuilt Valco valve. Analysis of SA was based on appropriate Multiple Reaction Monitoring (MRM) of ion pairs for labelled and endogenous SA using the following mass transitions: 2H_4_SA 141>97, SA 137>93. The MS was operated in negative ionization mode. The conditions were as follows: Temperature 500 °C, Ion source gas 1 50 psi, Ion source gas 2 60 psi, Ion Spray Voltage -4500 V, curtain gas 40 psi, Collision Gas Medium; DP (-25 V), EP (-9) and CXP (-2) were the same for all compounds. CE (-38), and DT (50) were used for 2H_4_SA and SA. Data were acquired and analysed using Analyst 1.4 software.

### *Pseudomonas syringae* infections

Plants were spray-inoculated with *Pseudomonas syringae DC3000* and *Pseudomonas syringae DC3000 hrcC-* at OD_600_ = 0.2 and sampled 2 h and 72 h after inoculation to determine the level of colonisation (colony-forming units (cfu)) as described previously [91]. In three biological replicates, a total of 30 plants were sampled for each plant genotype by each taking 3 leaf discs per plant.

### Quantitation of immunoblot membranes

Bradford assays were used to quantify protein levels in extracts and ensure equal loading of total proteins for gels used for immunoblot analysis and EMSA.

### Electro-mobility shift assay

Recombinant truncated protein of IDD4, IDD4-AA and IDD4-DD (each from amino acid 20–219) fused to MBP-tag was affinity-purified from *E*.*coli Rosetta* cells and enriched by Ion Exchange Purification.

3´ End-Biotinylated oligos were ordered. Biotinylated DNA (20fmol) was mixed with 1 μg of the indicated proteins after the instructions of the Lightshift Chemiluminescent DNA EMSA. These experiments were repeated at least three times with similar results.

### Kinase assays and phospho-site identification

Purified recombinant proteins and constitutively active MAPKs were mixed together in kinase reaction buffer (20 mM Tris-HCl pH 7.5, 10 mM MgCl_2_, 5 mM EGTA, 1 mM DTT and 50 μM ATP) and incubated at ambient temperature for 30 min. SDS-sample buffer was added to stop the reaction followed by boiling at 95°C for 10 min. Protein samples were resolved by SDS-PAGE. The gel was stained with SimplyBlue SafeStain and the band corresponding to the protein of interest was excised out, cut into small pieces of 0.5 mm3 and destained with four successive washes of 15 min each with ACN and 100 mM NH_4_HCO_3_. Proteins were reduced with 10 mM Tris(2-carboxyethyl)phosphine (TCEP) in 100 mM NH_4_HCO_3_ at 37°C for 1 h followed by alkylation with 20 mM S-Methyl methanethiosulfonate (MMTS) at ambient temperature for 30 min. Proteins were then digested with trypsin (Porcine trypsin) at 37°C overnight. The digestion was stopped by the addition of 1% formic acid, and the peptides were recovered by incubating the gel pieces in ACN. The recovered peptide solution was desalted using C18 ZipTip and analysed by LC-MS/MS. Briefly, peptide samples were separated on a C18 connected to an LTQ-Orbitrap Velos or a Q-Exactive HF instrument. The LC gradient ramped from 5% solvent B (water/ACN/formic acid, 20/80/0.1, v/v/v) to 45% solvent B over 45 min, then to 90% solvent B for 10 min. The MS instrument acquired fragmentation spectra on the top 10 peptides using CID fragmentation in the LTQ-Orbitrap or HCD in the Q-Exactive instrument. RAW data files obtained were converted to MGF files using Proteome Discoverer interface (version 1.4). Database searches were performed with the Mascot server v2.4 specifying the following parameters: database TAIR10 (release 2010/12/14, 35386 sequences); enzymatic specificity: trypsin permitting two allowed missed cleavages; fixed modification of cysteine residues (Methylthio(C)); possible variable modifications of phosphorylation on S, T and Y residues; 5 ppm tolerance on precursor masses and 0.5 Da tolerance on fragment ions. The results were filtered based on Mascot scores and MD-scores.

### Bimolecular fluorescence complementation (BiFC)

To obtain the expression vectors, coding sequences of candidate genes and MAPKs (kindly provided by J. Colcombet) were cloned in fusion with the N- and C-terminal parts of YFP, either as N- or C-terminal fusions, under the control of the cauliflower mosaic virus 35S (CaMV-35S) promoter in the pBIFC1,2,3 and 4 vectors [92]. Appropriate positive and negative controls were carried out for all combinations. Recombined vectors were individually transformed in *Agrobacterium tumefaciens* C58C1 strain by electroporation. Agrobacterium cultures from glycerol stocks were inoculated in 10 ml of LB medium with appropriate antibiotics and incubated for 24 h at 28°C with agitation. Each culture was pelleted and resuspended in infiltration buffer (10 mM MgCl_2_, 10 mM MES pH 5.7, 150 μM acetosyringone) to an OD_600_ of 1.5 and kept in the dark for 3 h. The P19 viral suppressor of gene silencing was co-expressed with each combination to prevent silencing of transiently expressed proteins [93]. 500 μl of each bacterial culture was mixed before infiltration. For fluorescence complementation, all eight possible combinations between a candidate gene and a MAPK were agro-infiltrated into 3-week-old *Nicotiana benthamiana* leaves. After 3 days, an upright confocal microscope with a 20X objective (Plan-Apochromat, NA 1.0) was used to visualise fluorescence. All images were acquired using Argon laser with 514-nm excitation.

### GUS staining

3 weeks old plants were incubated in GUS-Staining solution (NaPO_4_ (pH 7.2, 50mM), EDTA (pH8.0 10mM), TritonX100 (0.1%), Ferrocyanid (2mM), Ferricynaid (2mM), X-Gluc (1mg/ml)) for 12hr at 37°C. Subsequently, chlorophyll was bleached by applying 70% ETOH.

### DAB staining

Stainings were conducted by using 14-day old seedlings grown under sterile conditions on half MS medium, in accordance to [94].

### ROS burst assay

ROS burst assay was performed as described by [95].

### Accession numbers

*IDD4* (AT2G02080), *SAGT1* (AT2G43820), *MPK6* (AT2G43790).

## Supporting information

S1 Fig Expression analysis of *IDD4*. (**A,D,F**) *pIDD4*::*GUS* reporter lines driven by the 2.5kb upstream region of the translational start sequence of *IDD4* exhibit expression in trichomes (**A**), stomatas and epidermis cells (**D**), as well as in ovules (**F**). (**B,C,E**) *pIDD4*::*NLS*:*3xGFP* reporter line shows expression in the trichome (nucleus) (**B**), (**C**) shows the red channel auto-fluorescence of (**B**) and mesophyll cells (**E**). (**G**) Public microarray datasets accessible through Genevestigator platform revealed expression of *IDD4* during a wide range of tissues throughout the life cycle in *Arabidopsis*.
(PDF)

S2 Fig Characterization of *idd4* mutants, *IDD4ox* lines and transgenic lines expressing *IDD4* phospho-modified versions. **(A)** The *idd4* mutant allele resulting from the T-DNA insertion in the 1^st^ exon was confirmed to be homozygous by using the following primer combinations: 349as/350as (left border) on lane 1–4 and gene-specific primers 350s/349as on lane 5–8. Negative control, without DNA on lane 3 and 7. **(B)** Expression level of *IDD4* in *IDD4* over-expressor lines (*IDD4ox1-4*) compared to WT. **(C)** Phenotypic comparison of WT and transgenic *IDD4ox* lines grown on Murashige and Skoog basal medium under long-day conditions (12h day/12h night). **(D)** Shoot and root fresh weight of WT and 3 transgenic *IDD4ox* lines (*IDD4ox2-4*). Boxes represent the 25th and 75th percentiles and the inner rectangle highlights the median, whiskers show the SEM, letters above boxes represent significance groups as determined by multiple comparisons Student’s test *p*< 0.05. Plants of three biological replicates (n = 30) were analysed. **(E)** Fresh weight of shoot and root of 18 day-old WT plants compared to *idd4* complementation line (*pIDD4*::*IDD4*:*YFP*). Boxes represent the 25th and 75th percentiles and the inner rectangle highlights the median, whiskers show the SEM, letters above boxes represent significance groups as determined by multiple comparisons Student’s test *p*≤0.05. **(F)** Expression of SA-biosynthesis gene *CBP60g* and the defense-markers *WRKY22*, *PR2* and *FRK1* is elevated in *idd4* mutants. Error bars show ± SEM, statistical significance was analyzed by Student’s test against WT control *p<0.05. **(G)** Phenotypic comparison of 18 day-old seedlings of WT, *idd4*, *IDD4ox1*, *IDD4-AA and IDD4-DD* lines grown on Murashige and Skoog basal medium under long-day conditions (12h day/12h night). **(H-I)** Shoot **(H)** and root fresh weight **(I)** of 18 day-old WT, *idd4*, *IDD4ox*, *IDD4-AA* and *IDD4-DD* plants. Boxes represent the 25th and 75th percentiles and the inner rectangle highlights the median, whiskers show the SEM, letters above boxes represent significance groups as determined by multiple comparison Student’s test *p*< 0.01. Plants of three biological replicates (*n* = 30) were analysed.
(PDF)

S3 Fig IDD4 protein stability is not affected by flg22 perception. **(A)** GFP-antibody binding to GFP fusion protein of stable transgenic *IDD4*:*GFP Arabidopsis* lines used for ChIP-SEQ and ChIP-qPCR approach. Protein loading 30μg. **(B)** Binding profile of IDD4 for the *SCL3* locus. The TAIR annotation of the genomic locus is shown at the bottom of each panel. The genomic locus is in reverse orientation (-). The enrichment was found to be in the upstream region of the respective genomic locus (see also S3 Table). **(C)** Co-occurrence matrix illustrates overlapping of IDD4 binding to the same target sequences in the independent biological replicates. **(D)** Evaluation of *IDD4-AA*:*RFP* and *IDD4-DD*:*RFP* protein amount in stable transgenic *Arabidopsis* lines proven by RFP-antibody. Protein loading 30μg. **(E-F)** IDD4 protein stability is not affected after flg22 treatment as shown by Western-Blot analysis **(E)** and fluorescence microscopy **(F)** of 10 day-old seedlings of stable transgenic lines.
(PDF)

S4 Fig Schematic representation of IDD4 amino acid sequence and interaction of IDD4 and phospho-modified versions with known binding partners. **(A)** Depiction of IDD4 amino acid sequence. Phosphorylation sites are highlighted in red. The ZFs are highlighted in orange. The MAPK docking motif[KR]{0,2}[KR].{0,2}[KR].{2,4}[ILVM].[ILVF] (*p* = 4.324e^-03^) is underlined in red. **(B-D)** The expression of the defense marker *PR1* **(B)** is up-regulated in the *IDD4-AA* line 4 hrs after flg22-treatment compared to WT and *IDD4-DD* line. The SA-signaling inhibitors *NIMIN1* **(C)** and *WRKY38* **(D)** are up-regulated in *IDD4-DD* 4 hrs after flg22 treatment and *NIMIN1* is diminished in the *IDD4-AA* line. Error bars show ± SEM, statistical significance was analyzed by Student’s test, letters above bars represent significance groups, *p*<0.01. **(E)** Yeast two-hybrid interactions of IDD4, IDD4-AA and IDD4-DD with the *Arabidopsis* DELLA protein GAI. Growth on selective plates lacking leucine, tryptophan, adenine and histidine (SD-LWAH) and on control plates lacking leucine and tryptophan (SD-LW) is shown. **(F)** BiFC interaction of IDD4-AA and IDD4-DD with the *Arabidopsis* SCL3 protein after transient expression in tobacco leaves. Scale bar = 50 μm.
(PDF)

S5 Fig Protein interaction networks derived from the IDD4 ChIP-SEQ targets that are concomitantly differentially regulated in *idd4* mutant and/ or *IDD4ox* lines. **(A)** Protein interaction network of IDD4 ChIP-SEQ targets being prevalently up-regulated in *idd4* mutant and down-regulated in *IDD4o*x lines. **(B)** Protein interaction network of IDD4 ChIP-SEQ targets being prevalently down-regulated in *idd4* mutant and up-regulated in *IDD4o*x lines. All significant targets were pooled and used to generate a network using STRING (version 10.0) followed by network clustering. Minimum required interaction score defined as medium confidence, Meaning of network edges “evidence”.
(PDF)

S1 Table Heatmap matrix derived from the transcriptome comparison of WT and *idd4* mutant untreated and after flg22 treatment. The original FPKM values are depicted that were adjusted by normalized genes/rows and subsequently processed by hierarchical clustering by average linkage method using MeV4.0.
(XLSX)

S2 Table RNA-Hiseq and gene-ontology analysis are presented of *idd4* (mock/flg22 treatment 1hr) and *IDD4* over-expressor line (*IDD4ox1*). Furthermore, genes oppositely differentially expressed in *idd4* vs. *IDD4ox* are listed.
(XLSX)

S3 Table Presentation of the IDD4 ChIP-SEQ target list and the dedicated gene ontology terms. Furthermore, common target genes are presented of RGA and IDD4 ChIP-SEQ study.
(XLSX)

S4 Table ChIP-SEQ targets are listed that are predominantly oppositely deregulated in *idd4* mutant and *IDD4ox* lines associated with STRING-based cluster analysis. (XLSX)

S5 Table *In silico* analysis of the genome-wide 500 bp upstream sequence of the TSS at the occurrence of at least two *ID1*-core motifs. (XLSX)

S6 Table GO term analysis of significantly differentially regulated genes in IDD4-AA and IDD4-DD lines compared to WT. Overview of number of differentially expressed genes among *idd4* mutant, *IDD4-AA*, *IDD4-DD* and *IDD4ox* plants.
(XLSX)

S7 Table Overview of used oligo-nucleotides. (XLSX)
