# Supplementary material for: INDETERMINATE-DOMAIN 4 (IDD4) coordinates immune responses with plant-growth in Arabidopsis thaliana
Source: PLoS Pathog. 2019 Jan 24;15(1):e1007499. doi: 10.1371/journal.ppat.1007499 (PMC6345439; doi:10.1371/journal.ppat.1007499)
Supplement: S1 Fig — (A,D,F) pIDD4::GUS reporter lines driven by the 2.5kb upstream region of the translational start sequence of IDD4 exhibit expression in trichomes (A), stomatas and epidermis cells (D), as well as in ovules (F). (B,C,E) pIDD4::NLS:3xGFP reporter line shows expression in the trichome (nucleus) (B), (C) shows the red channel auto-fluorescence of (B) and mesophyll cells (E). (G) Public microarray datasets accessible through Genevestigator platform revealed expression of IDD4 during a wide range of tissues throughout the life cycle in Arabidopsis. (PDF) [file ppat.1007499.s001.pdf]

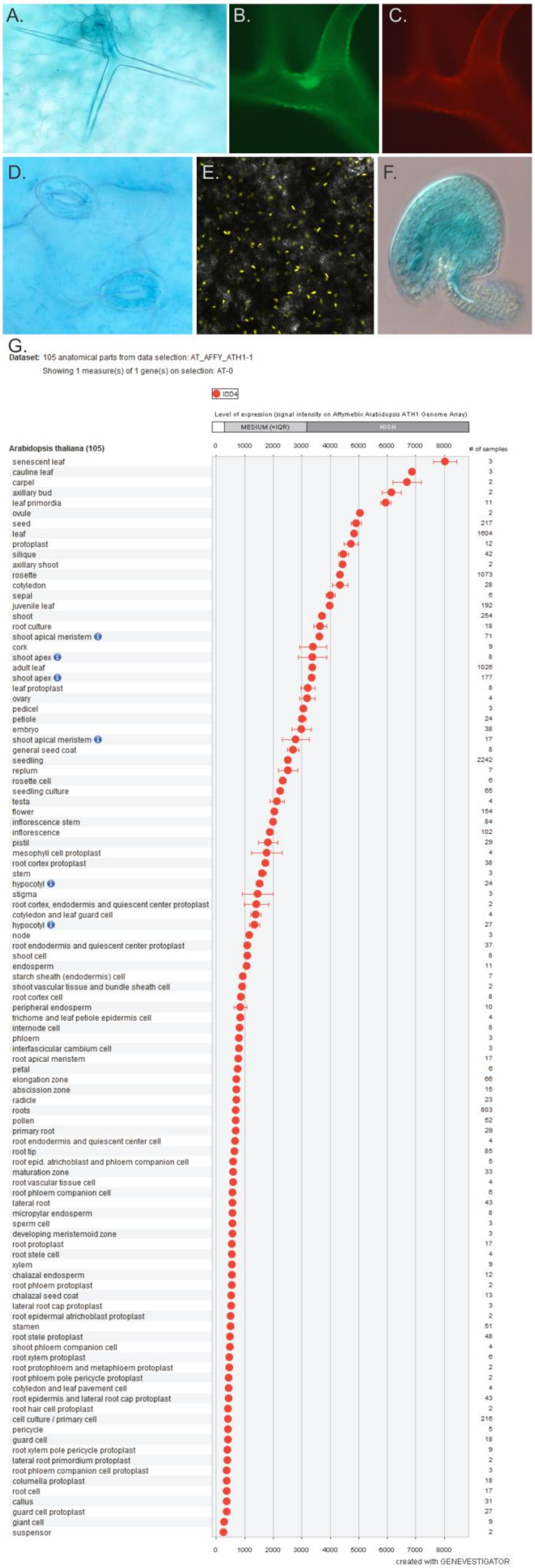

S1 Fig. Expression analysis of *IDD4*

(**A,D,F**) *pIDD4::GUS* reporter lines driven by the 2.5kb upstream region of the translational start sequence of *IDD4* exhibit expression in trichomes (**A**), stomatas and epidermis cells (**D**), as well as in ovules (**F**).
(**B,C,E**) *pIDD4::NLS:3xGFP* reporter line shows expression in the trichome (nucleus) (**B**),
(**C**) shows the red channel auto-fluorescence of (**B**) and mesophyll cells (**E**).
(**G**) Public microarray datasets accessible through Genevestigator platform revealed expression of *IDD4* during a wide range of tissues throughout the life cycle in *Arabidopsis*.
