# Supplementary material for: INDETERMINATE-DOMAIN 4 (IDD4) coordinates immune responses with plant-growth in Arabidopsis thaliana
Source: PLoS Pathog. 2019 Jan 24;15(1):e1007499. doi: 10.1371/journal.ppat.1007499 (PMC6345439; doi:10.1371/journal.ppat.1007499)
Supplement: S2 Fig — (A) The idd4 mutant allele resulting from the T-DNA insertion in the 1st exon was confirmed to be homozygous by using the following primer combinations: 349as/350as (left border) on lane 1–4 and gene-specific primers 350s/349as on lane 5–8. Negative control, without DNA on lane 3 and 7. (B) Expression level of IDD4 in IDD4 over-expressor lines (IDD4ox1-4) compared to WT. (C) Phenotypic comparison of WT and transgenic IDD4ox lines grown on Murashige and Skoog basal medium under long-day conditions (12h day/12h night). (D) Shoot and root fresh weight of WT and 3 transgenic IDD4ox lines (IDD4ox2-4). Boxes represent the 25th and 75th percentiles and the inner rectangle highlights the median, whiskers show the SEM, letters above boxes represent significance groups as determined by multiple comparisons Student’s test p< 0.05. Plants of three biological replicates (n = 30) were analysed. (E) Fresh weight of shoot and root of 18 day-old WT plants compared to idd4 complementation line (pIDD4::IDD4:YFP). Boxes represent the 25th and 75th percentiles and the inner rectangle highlights the median, whiskers show the SEM, letters above boxes represent significance groups as determined by multiple comparisons Student’s test p≤0.05. (F) Expression of SA-biosynthesis gene CBP60g and the defense-markers WRKY22, PR2 and FRK1 is elevated in idd4 mutants. Error bars show ± SEM, statistical significance was analyzed by Student’s test against WT control *p<0.05. (G) Phenotypic comparison of 18 day-old seedlings of WT, idd4, IDD4ox1, IDD4-AA and IDD4-DD lines grown on Murashige and Skoog basal medium under long-day conditions (12h day/12h night). (H-I) Shoot (H) and root fresh weight (I) of 18 day-old WT, idd4, IDD4ox, IDD4-AA and IDD4-DD plants. Boxes represent the 25th and 75th percentiles and the inner rectangle highlights the median, whiskers show the SEM, letters above boxes represent significance groups as determined by multiple comparison Student’s test p< 0.01. Plants of three biol [file ppat.1007499.s002.pdf]

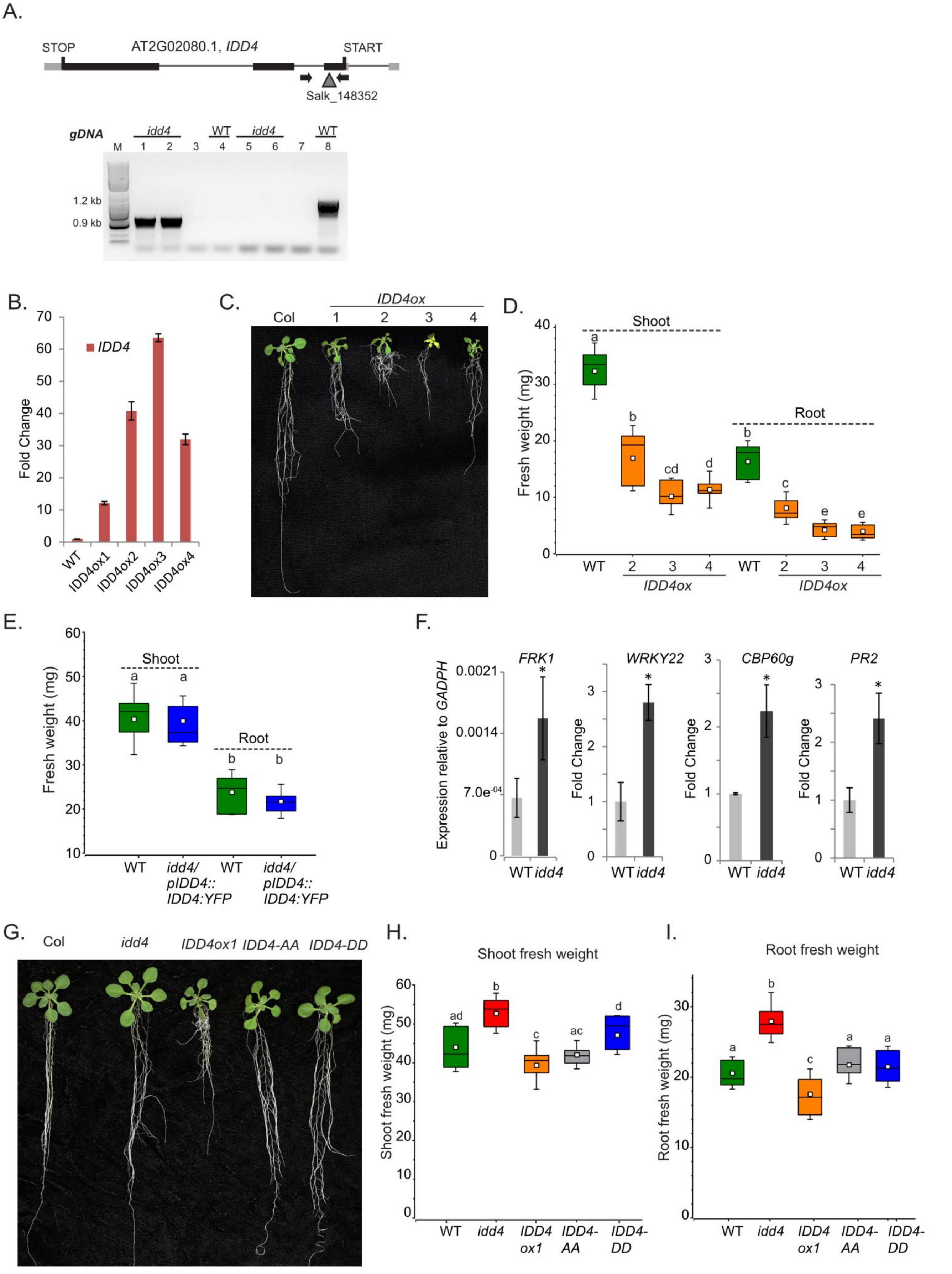
A.
STOP
AT2G02080.1, IDD4
START
Salk_148352
gDNA
idd4
WT
idd4
WT
M 1 2 3 4 5 6 7 8
1.2 kb
0.9 kb
B.
IDD4
Fold Change
70
60
50
40
30
20
10
0
WT
IDD4ox1
IDD4ox2
IDD4ox3
IDD4ox4
C.
IDD4ox
Col 1 2 3 4
D.
Shoot
Root
Fresh weight (mg)
40
30
20
10
0
a
b
cd
d
b
c
e
e
WT 2 3 4 WT 2 3 4
IDD4ox
IDD4ox
E.
Fresh weight (mg)
60
50
40
30
20
10
Shoot
Root
a
a
b
b
WT
idd4/ pIDD4:: IDD4:YFP
WT
idd4/ pIDD4:: IDD4:YFP
F.
FRK1
WRKY22
CBP60g
PR2
Expression relative to GADPH
0.0021
0.0014
7.0e-04
0
Fold Change
*
WT idd4
G.
Col
idd4
IDD4ox1
IDD4-AA
IDD4-DD
H.
Shoot fresh weight
Shoot fresh weight (mg)
60
50
40
30
20
10
0
ad
b
c
ac
d
WT
idd4
IDD4 ox1
IDD4- AA
IDD4- DD
I.
Root fresh weight
Root fresh weight (mg)
30
20
10
0
a
b
c
a
a
WT
idd4
IDD4 ox1
IDD4- AA
IDD4- DD

S2 Fig. Characterization of *idd4* mutants, *IDD4ox* lines and transgenic lines expressing *IDD4* phospho-modified versions

(A) The *idd4* mutant allele resulting from the T-DNA insertion in the 1st exon was confirmed to be homozygous by using the following primer combinations: 349as/350as (left border) on lane 1-4 and gene-specific primers 350s/349as on lane 5-8. Negative control, without DNA on lane 3 and 7.

(B) Expression level of *IDD4* in *IDD4* overexpressor lines (*IDD4ox1-4*) compared to WT.

(C) Phenotypic comparison of WT and transgenic *IDD4ox* lines grown on Murashige and Skoog basal medium under long-day conditions (12h day/12h night).

(D) Shoot and root fresh weight of WT and 3 transgenic *IDD4ox* lines (*IDD4ox2-4*). Boxes represent the 25th and 75th percentiles and the inner rectangle highlights the median, whiskers show the SEM, letters above boxes represent significance groups as determined by multiple comparison Student's test $p < 0.05$. Plants of three biological replicates (n=30) were analysed.

(E) Fresh weight of shoot and root of 18 day-old WT plants compared to *idd4* complementation line (*pIDD4::IDD4:YFP*). Boxes represent the 25th and 75th percentiles and the inner rectangle highlights the median, whiskers show the SEM, letters above boxes represent significance groups as determined by multiple comparison Student's test $p \leq 0.05$.

(F) Expression of SA-biosynthesis gene *CBP60g* and the defense-markers *WRKY22*, *PR2* and *FRK1* is elevated in *idd4* mutants. Error bars show ± SEM, statistical significance was analyzed by Student's test against WT control *$p < 0.05$.

(G) Phenotypic comparison of 18 day-old seedlings of WT, *idd4, IDD4ox1, IDD4-AA and IDD4-DD* lines grown on Murashige and Skoog basal medium under long-day conditions (12h day/12h night).

(H-I) Shoot **(H)** and root fresh weight **(I)** of 18 day-old WT, *idd4*, *IDD4ox*, *IDD4-AA* and *IDD4-DD* plants. Boxes represent the 25th and 75th percentiles and the inner rectangle highlights the median, whiskers show the SEM, letters above boxes represent significance groups as determined by multiple comparison Student's test $p < 0.01$. Plants of three biological replicates (n=30) were analysed.
