# Supplementary material for: INDETERMINATE-DOMAIN 4 (IDD4) coordinates immune responses with plant-growth in Arabidopsis thaliana
Source: PLoS Pathog. 2019 Jan 24;15(1):e1007499. doi: 10.1371/journal.ppat.1007499 (PMC6345439; doi:10.1371/journal.ppat.1007499)
Supplement: S3 Fig — (A) GFP-antibody binding to GFP fusion protein of stable transgenic IDD4:GFP Arabidopsis lines used for ChIP-SEQ and ChIP-qPCR approach. Protein loading 30μg. (B) Binding profile of IDD4 for the SCL3 locus. The TAIR annotation of the genomic locus is shown at the bottom of each panel. The genomic locus is in reverse orientation (-). The enrichment was found to be in the upstream region of the respective genomic locus (see also S3 Table). (C) Co-occurrence matrix illustrates overlapping of IDD4 binding to the same target sequences in the independent biological replicates. (D) Evaluation of IDD4-AA:RFP and IDD4-DD:RFP protein amount in stable transgenic Arabidopsis lines proven by RFP-antibody. Protein loading 30μg. (E-F) IDD4 protein stability is not affected after flg22 treatment as shown by Western-Blot analysis (E) and fluorescence microscopy (F) of 10 day-old seedlings of stable transgenic lines. (PDF) [file ppat.1007499.s003.pdf]

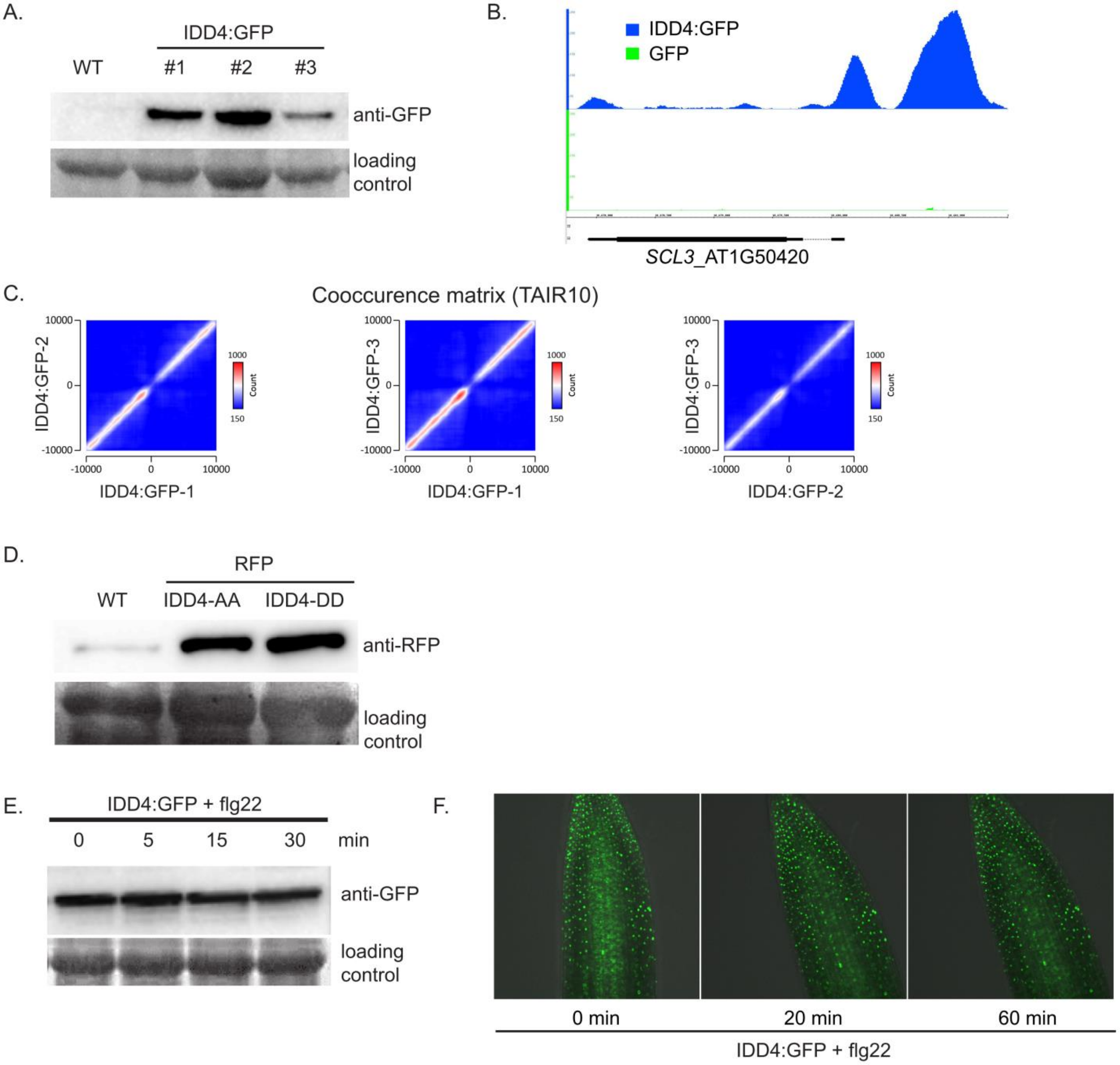

S3 Fig. IDD4 protein stability is not affected by flg22 perception

(A) GFP-antibody binding to GFP fusion protein of stable transgenic *IDD4:GFP Arabidopsis* lines used for ChIP-SEQ and ChIP-qPCR approach. Protein loading 30µg.
(B) Binding profile of IDD4 for the *SCL3* locus. The TAIR annotation of the genomic locus is shown at the bottom of each panel. The genomic locus is in reverse orientation (-). The enrichment was found to be in the upstream region of the respective genomic locus (see also **Table S3**).
(C) Co-occurrence matrix illustrates overlapping of IDD4 binding to the same target sequences in the independent biological replicates.
(D) Evaluation of *IDD4-AA:RFP* and *IDD4-DD:RFP* protein amount in stable transgenic *Arabidopsis* lines proven by RFP-antibody. Protein loading 30µg.
(E-F) IDD4 protein stability is not affected after flg22 treatment as shown by Western-Blot analysis **(E)** and fluorescence microscopy **(F)** of 10 day-old seedlings of stable transgenic lines.
