# Supplementary material for: INDETERMINATE-DOMAIN 4 (IDD4) coordinates immune responses with plant-growth in Arabidopsis thaliana
Source: PLoS Pathog. 2019 Jan 24;15(1):e1007499. doi: 10.1371/journal.ppat.1007499 (PMC6345439; doi:10.1371/journal.ppat.1007499)
Supplement: S4 Fig — (A) Depiction of IDD4 amino acid sequence. Phosphorylation sites are highlighted in red. The ZFs are highlighted in orange. The MAPK docking motif[KR]{0,2}[KR].{0,2}[KR].{2,4}[ILVM].[ILVF] (p = 4.324e-03) is underlined in red. (B-D) The expression of the defense marker PR1 (B) is up-regulated in the IDD4-AA line 4 hrs after flg22-treatment compared to WT and IDD4-DD line. The SA-signaling inhibitors NIMIN1 (C) and WRKY38 (D) are up-regulated in IDD4-DD 4 hrs after flg22 treatment and NIMIN1 is diminished in the IDD4-AA line. Error bars show ± SEM, statistical significance was analyzed by Student’s test, letters above bars represent significance groups, p<0.01. (E) Yeast two-hybrid interactions of IDD4, IDD4-AA and IDD4-DD with the Arabidopsis DELLA protein GAI. Growth on selective plates lacking leucine, tryptophan, adenine and histidine (SD-LWAH) and on control plates lacking leucine and tryptophan (SD-LW) is shown. (F) BiFC interaction of IDD4-AA and IDD4-DD with the Arabidopsis SCL3 protein after transient expression in tobacco leaves. Scale bar = 50 μm. (PDF) [file ppat.1007499.s004.pdf]

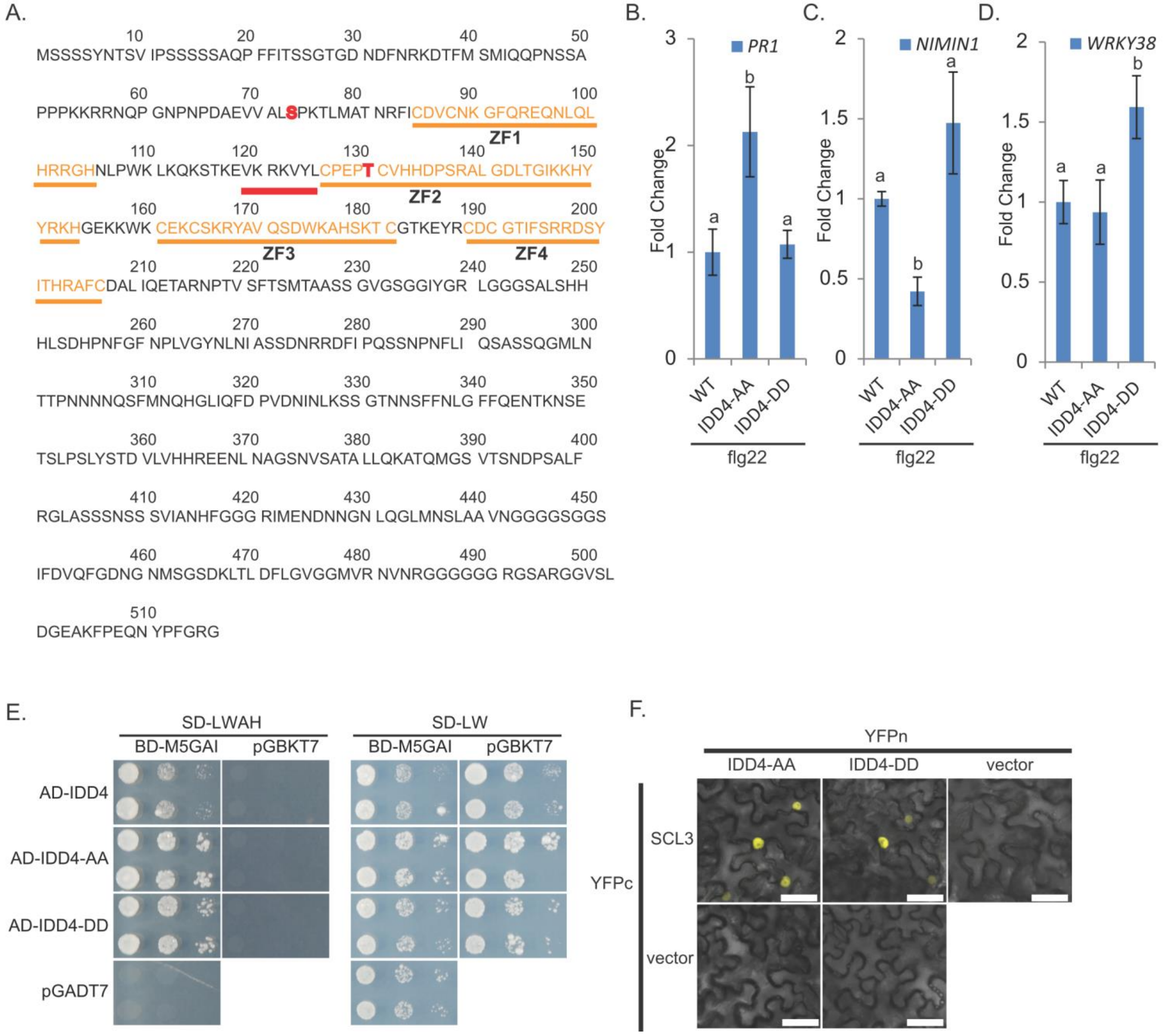

S4 Fig. Schematic representation of IDD4 amino acid sequence and interaction of IDD4 and phospho-modified versions with known binding partners

(A) Depiction of IDD4 amino acid sequence. Phosphorylation sites are highlighted in red. The ZFs are highlighted in orange. The MAPK docking motif[KR]{0,2}[KR].{0,2}[KR].{2,4}[ILVM].[ILVF] (p=4.324e^{-03}) is underlined in red.

(B-D) The expression of the defense marker *PR1* **(B)** is up-regulated in the *IDD4-AA* line 4 hrs after flg22-treatment compared to WT and *IDD4-DD* line. The SA-signaling inhibitors *NIMIN1* **(C)** and *WRKY38* **(D)** are up-regulated in *IDD4-DD* 4 hrs after flg22 treatment and *NIMIN1* is diminished in the *IDD4-AA* line. Error bars show ± SEM, statistical significance was analyzed by Student's test, letters above bars represent significance groups, p<0.01.

(E) Yeast two-hybrid interactions of IDD4, IDD4-AA and IDD4-DD with the *Arabidopsis* DELLA protein GAI. Growth on selective plates lacking leucine, tryptophan, adenine and histidine (SD-LWAH) and on control plates lacking leucine and tryptophan (SD-LW) is shown.

(F) BiFC interaction of IDD4-AA and IDD4-DD with the *Arabidopsis* SCL3 protein after transient expression in tobacco leaves. Scale bar=50 µm.
