# Supplementary material for: INDETERMINATE-DOMAIN 4 (IDD4) coordinates immune responses with plant-growth in Arabidopsis thaliana
Source: PLoS Pathog. 2019 Jan 24;15(1):e1007499. doi: 10.1371/journal.ppat.1007499 (PMC6345439; doi:10.1371/journal.ppat.1007499)
Supplement: S5 Fig — (A) Protein interaction network of IDD4 ChIP-SEQ targets being prevalently up-regulated in idd4 mutant and down-regulated in IDD4ox lines. (B) Protein interaction network of IDD4 ChIP-SEQ targets being prevalently down-regulated in idd4 mutant and up-regulated in IDD4ox lines. All significant targets were pooled and used to generate a network using STRING (version 10.0) followed by network clustering. Minimum required interaction score defined as medium confidence, Meaning of network edges “evidence”. (PDF) [file ppat.1007499.s005.pdf]

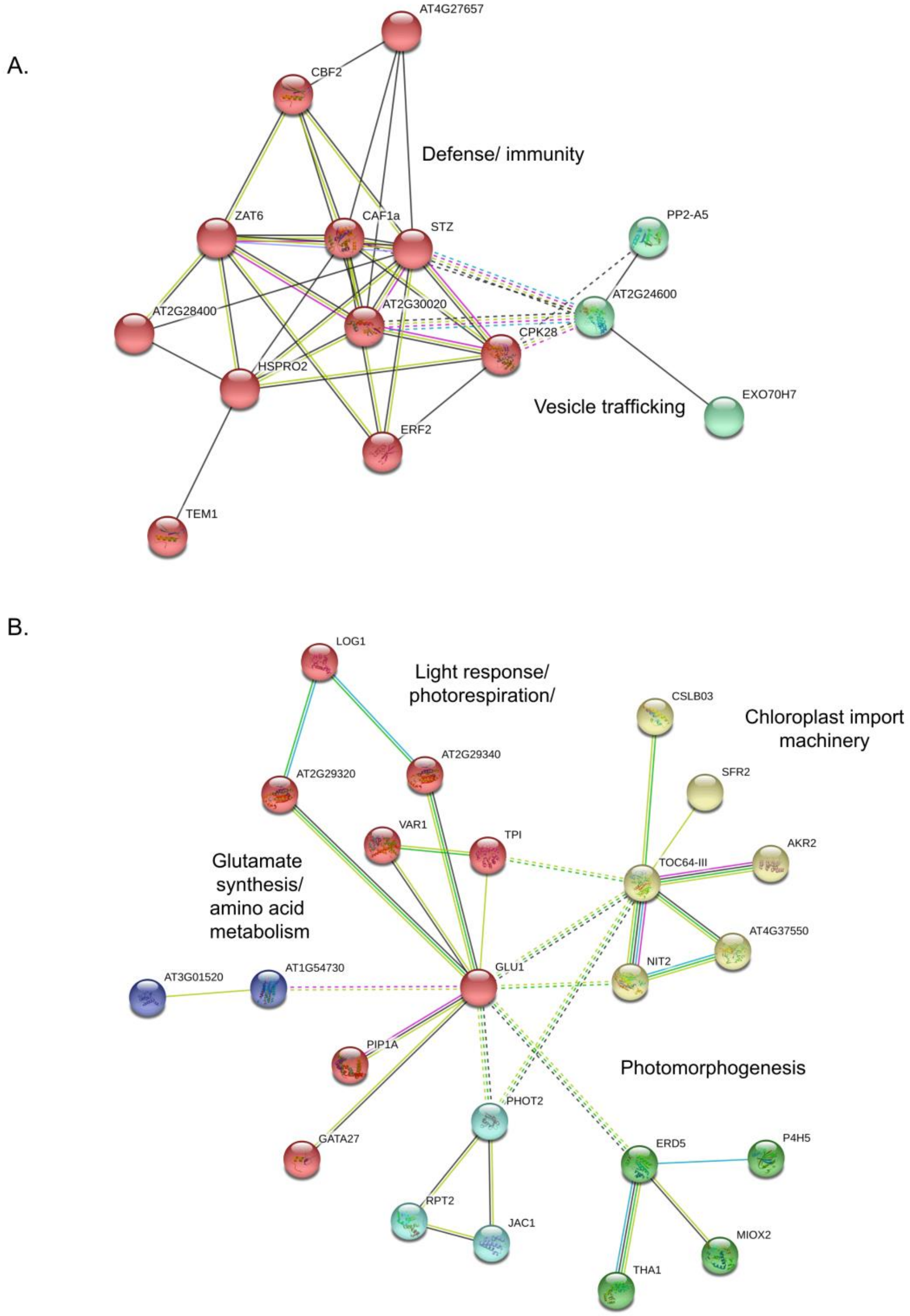

S5 Fig. Protein interaction networks derived from the IDD4 ChIP-SEQ targets that are concomitantly differentially regulated in *idd4* mutant and/ or *IDD4ox* lines.

(A) Protein interaction network of IDD4 ChIP-SEQ targets being prevalently up-regulated in *idd4* mutant and down-regulated in *IDD4o*x lines.
(B) Protein interaction network of IDD4 ChIP-SEQ targets being prevalently down-regulated in *idd4* mutant and up-regulated in *IDD4o*x lines.
All significant targets were pooled and used to generate a network using STRING (version 10.0) followed by network clustering. Minimum required interaction score defined as medium confidence, Meaning of network edges “evidence”.
